# “Low-” versus “high”-frequency oscillation and right ventricular function in ARDS. A randomized crossover study

**DOI:** 10.1186/s40560-018-0327-3

**Published:** 2018-09-04

**Authors:** Spyros D. Mentzelopoulos, Hector Anninos, Sotirios Malachias, Spyros G. Zakynthinos

**Affiliations:** First Department of Intensive Care Medicine, National and Kapodestrian University of Athens Medical School, Evaggelismos General Hospital, 45-47 Ipsilandou Street, GR-10675 Athens, Greece

**Keywords:** High-frequency ventilation, Respiratory distress syndrome, Adult, Heart ventricles, Hypercapnia, Echocardiography, Transesophageal

## Abstract

**Background:**

Recent, large trials of high-frequency oscillation (HFO) versus conventional ventilation (CV) in acute respiratory distress syndrome (ARDS) reported negative results. This could be explained by an HFO-induced right ventricular (RV) dysfunction/failure due to high intrathoracic pressures and hypercapnia. We hypothesized that HFO strategies aimed at averting/attenuating hypercapnia, such as “low-frequency” (i.e., 4 Hz) HFO and 4-Hz HFO with tracheal-gas insufflation (HFO-TGI), may result in an improved RV function relative to “high-frequency” (i.e., 7 Hz) HFO (which may promote hypercapnia) and similar RV function relative to lung protective CV.

**Methods:**

We studied 17 patients with moderate-to-severe ARDS [PaO_2_-to-inspiratory O_2_ fraction ratio (PaO_2_/FiO_2_) < 150]. RV function was assessed by transesophageal echocardiography (TEE). Patients received 60 min of CV for TEE-guided, positive end-expiratory pressure (PEEP) “optimization” and subsequent stabilization; 60 min of 4-Hz HFO for “study mean airway pressure (mPaw)” titration to peripheral oxygen saturation ≥ 95%, without worsening RV function as assessed by TEE; 60 min of each tested HFO strategy in random order; and another 60 min of CV using the pre-HFO, TEE-guided PEEP setting. Study measurements (i.e., gas exchange, hemodynamics, and TEE data) were obtained over the last 10 min of pre-HFO CV, of each one of the three tested HFO strategies, and of post-HFO CV.

**Results:**

The mean “study HFO mPaw” was 8–10 cmH_2_O higher relative to pre-HFO CV. Seven-Hz HFO versus 4-Hz HFO and 4-Hz HFO-TGI resulted in higher mean ± SD right-to-left ventricular end-diastolic area ratio (RVEDA/LVEDA) (0.64 ± 0.15 versus 0.56 ± 0.14 and 0.52 ± 0.10, respectively, both *p* < 0.05). Higher diastolic/systolic eccentricity indexes (1.33 ± 0.19/1.42 ± 0.17 versus 1.21 ± 0.10/1.26 ± 0.10 and 1.17 ± 0.11/1.17 ± 0.13, respectively, all *p* < 0.05). Seven-Hz HFO resulted in 18–28% higher PaCO_2_ relative to all other ventilatory strategies (all *p* < 0.05). Four-Hz HFO-TGI versus pre-HFO CV resulted in 15% lower RVEDA/LVEDA, and 7%/10% lower diastolic/systolic eccentricity indexes (all *p* < 0.05). Mean PaO_2_/FiO_2_ improved by 77–80% during HFO strategies versus CV (all *p* < 0.05). Mean cardiac index varied by ≤ 10% among strategies. Percent changes in PaCO_2_ among strategies were predictive of concurrent percent changes in measures of RV function (*R*^2^ = 0.21–0.43).

**Conclusions:**

In moderate-to-severe ARDS, “short-term” 4-Hz HFO strategies resulted in better RV function versus 7-Hz HFO, partly attributable to improved PaCO_2_ control, and similar or improved RV function versus CV.

**Trial registration:**

This study was registered 40 days prior to the enrollment of the first patient at ClinicalTrials.gov, ID no. NCT02027129, Principal Investigator Spyros D. Mentzelopoulos, date of registration January 3, 2014.

**Electronic supplementary material:**

The online version of this article (10.1186/s40560-018-0327-3) contains supplementary material, which is available to authorized users.

## Background

High-frequency oscillation (HFO) is a ventilatory strategy employing tidal volumes (Vts) of < 4 mL/kg predicted body weight administered at frequencies of 3–15 Hz [1]. Despite previous encouraging findings [2–6], two recently published, multicenter, randomized clinical trials (RCTs) that compared HFO with conventional ventilation (CV) in early acute respiratory distress syndrome (ARDS) reported either neutral results [7] or an HFO-induced harm [8]. These RCTs employed “high-frequency” (i.e., mean, day 1 and day 2 frequency settings of 5.5–7.8 and 6.6–7.5 Hz, respectively) HFO without tracheal tube cuff leak and CV with moderate or low-Vt and moderate or high-positive end-expiratory pressure (PEEP) [7, 8]. These results might be partly attributable to right ventricular (RV) overload, dysfunction, and failure caused by concurrent high intrathoracic pressures and hypercapnia, and consequent hemodynamic instability and increased need for inotropic/vasopressor support [9–12]. Indeed, mortality rate is increased in ARDS patients with acute cor pulmonale [13].

Notably, prior two-center data on moderate-to-severe ARDS suggested a survival benefit from the intermittent use of “low-frequency” (i.e., approximately 4 Hz) HFO with tracheal tube cuff leak and tracheal gas insufflation (TGI) [14]. “Low”-HFO frequency, cuff leak, and TGI augment CO_2_ elimination [14, 15], with potential benefit on RV function.

The development of lung protective CV aimed at minimizing lung injury and organ dysfunction [16] was based on decades of laboratory and clinical research. This research subsequently paved the way for the conception and conduct of the conclusive ARDSnet trial [17]. In sharp contrast, recently proposed and tested HFO strategies [7, 8] were based just on pilot clinical data and expert consensus [8]; furthermore, these HFO strategies were focused at preventing lung injury, without concurrently protecting the RV [7–12, 18]. Therefore, discrepant prior data [6, 14] and recent RCT results [7, 8] might partly reflect differences in protocol-employed HFO ventilator settings and their impact on RV function and patient outcomes.

In the present physiological study, we used transesophageal echocardiography (TEE) and tested the hypothesis that “short-term” “low-frequency” HFO with cuff leak and with or without TGI,—i.e., HFO strategies aimed at preventing hypercapnia [14, 15]—might result in (1) improved RV function as compared with a “high-frequency” HFO strategy without cuff leak or TGI, which may promote hypercapnia [7, 8], and (2) similar RV function relative to lung protective CV. Avoiding excessive hypercapnia might prevent or attenuate RV dysfunction/failure during the application of high-HFO mean airway pressure (mPaw) aimed at augmenting lung recruitment [9–13].

## Methods

Additional details, including pre-study patient preparation, are presented in Additional file 1.

### Research ethics committee approval and informed consent

This study was approved by the Evaggelismos Hospital Scientific Committee (approval no. 271-30-10-2013), and written informed consent was obtained from the next-of-kin of all participating patients. The study was registered 40 days prior to the enrollment of the first patient at ClinicalTrials.gov (NCT02027129, Principal Investigator Spyros D. Mentzelopoulos, date of registration January 3, 2014).

### Patients

**Fig. 1:**
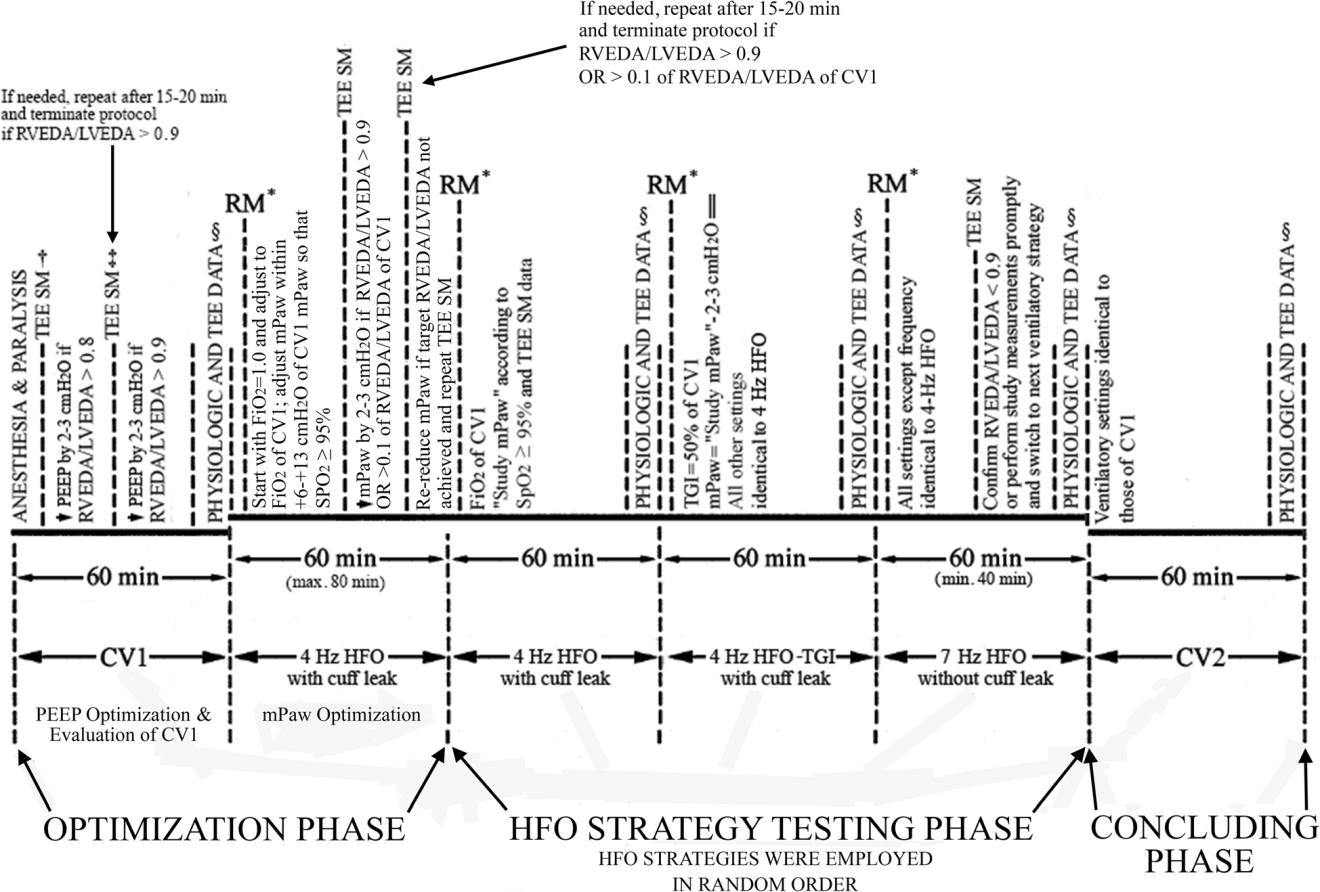
Schematic representation of the study protocol. CV1 first period of lung protective conventional ventilation, PEEP positive end-expiratory pressure, TEE transesophageal echocardiography, SM safety measurement, RVEDA right ventricular end-diastolic area, LVEDA left ventricular end-diastolic area, FiO_2_ inspiratory oxygen fraction, RM recruitment maneuver, mPaw mean airway pressure, SpO_2_ peripheral oxygen saturation, HFO high-frequency oscillation, TGI tracheal gas insufflation, CV2 second (study protocol concluding) period of lung-protective conventional ventilation. *Continuous positive airway pressure of 45 cmH_2_O for 40 s; the HFO breathing circuit was pressurized with the oscillator piston off. ^†^During the first study TEE SM (measurement duration, < 5 min), an RVEDA/LVDEA ratio of > 0.8 triggered a PEEP decrease by 2–3 cmH_2_O. ^‡^Performed within 15–20 min after a protocol-mandated decrease in PEEP (see above); this (second) TEE SM was not performed whenever RVEDA/LVEDA did not exceed 0.8 at the first TEE SM. ^§^Includes saved midesophageal four-chamber views and, transgastric, two-chamber, short-axis views, and hemodynamic and gas exchange data during all ventilator strategy testing periods; quasistatic respiratory compliance data were also obtained during CV1 and CV2; each time, the study protocol measurements were to be completed within 10 min. ^║^During HFO-TGI, the mPaw was set at 2–3 cmH_2_O lower than the “study HFO mPaw” to counterbalance an estimated, TGI-associated increase of similar magnitude in HFO tracheal pressure; see also text, reference [15] and Additional file 1

We studied patients with early (onset within preceding 72 h) ARDS and a PaO_2_-to-inspiratory O_2_ fraction (FiO_2_) ratio of < 150. Patients were for ≥ 24 h on low-Vt CV [14, 17] with FiO_2_ set within 0.50–0.90 and PEEP set within 10–17 cmH_2_O. Eleven patients had moderate ARDS and 6 patients had severe ARDS according to the Berlin definition [19]. Patients were nursed in the 27-bed Intensive Care Unit of Evaggelismos Hospital, Athens, Greece. Study eligibility criteria are presented in Additional file 1: Table S1. The departmental, lung protective CV protocol is presented in Additional file 1: Table S2. Figure 1 displays a study protocol schema. Continuous patient monitoring comprised electrocardiographic lead II, peripheral oxygen saturation (SpO_2_), intraarterial pressure, and cardiac index by pulse-induced contour cardiac output (PiCCO) *Plus* (PICCO Plus, Pulsion Medical Systems) [14, 15]. Patients were anesthetized and paralyzed throughout the study [15].

### Study protocol

#### Pre-HFO CV—TEE guided setting of PEEP—part 1 of optimization phase (Fig. 1)

**Fig. 2:**
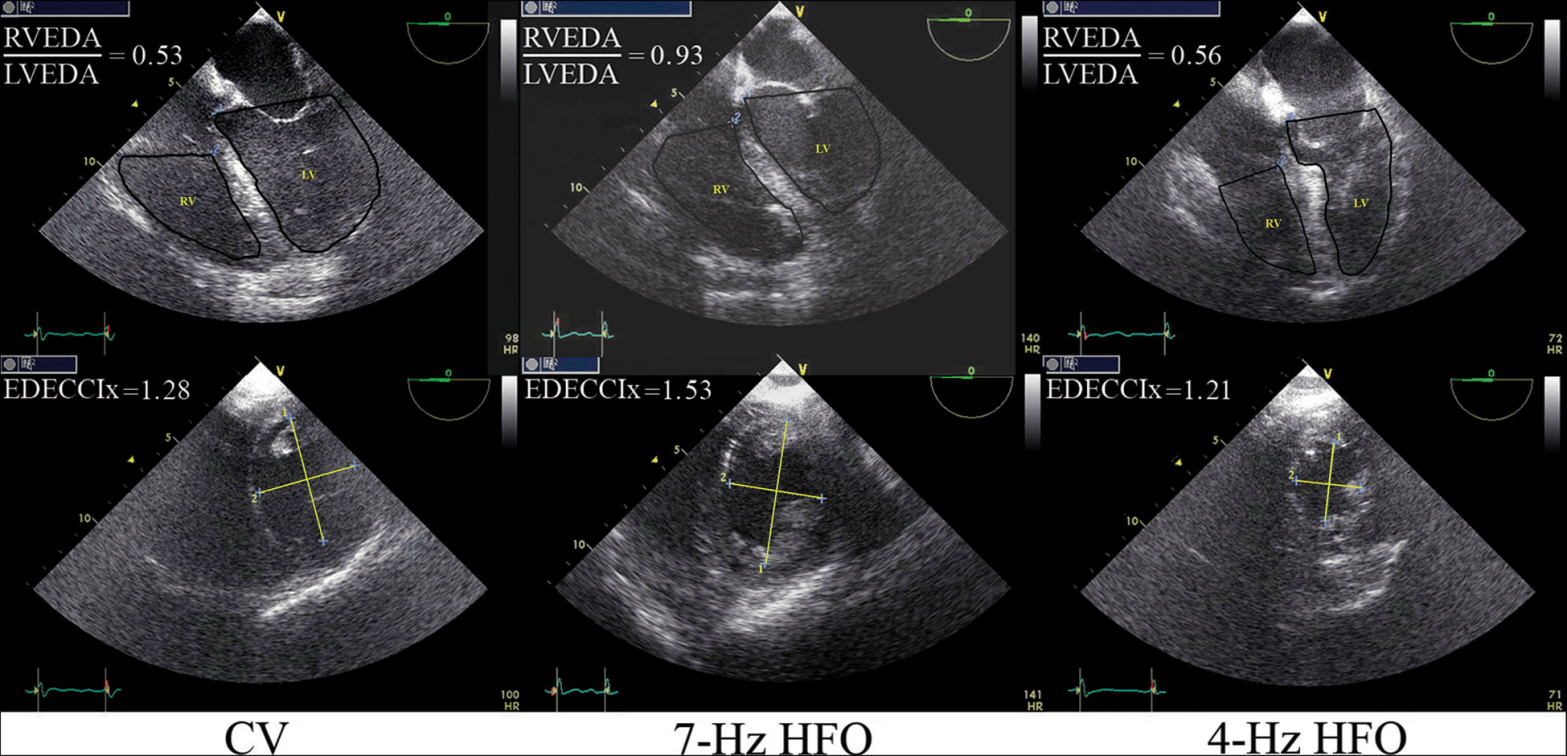
Examples of transesophageal echocardiographic (TEE) determination of two primary study outcome variables. Upper panel: determination of the right-to-left ventricular end-diastolic area ratio (RVEDA/LVEDA). Lower panel: determination of the end-diastolic eccentricity index (EDECCIx). The left vertical pair of images was obtained from study participant no. 12 during conventional ventilation (CV), the middle pair of images was obtained from participant no. 7 during 7-Hz high-frequency oscillation (HFO), and the right pair of images was obtained from participant no. 13 during 4-Hz HFO. In the lower panel (short-axis, transgastric views), “2” corresponds to the diameter of the left ventricle (LV) that was perpendicular to and bisecting the interventricular septum, whereas “1” corresponds to the diameter that was perpendicular to “2.” TEE measurements were repeated and averaged over 2–4 consecutive cardiac cycles (see also Additional file 1); EDECCIx was calculated as diameter “1”/diameter “2.” Description of figure modifications performed with Photoshop CC (Adobe Systems): upper panel, enhancement of the periphery of the right ventricle (RV) and LV using the “pen” tool and the “stroke path” command and enhancement of contrast of the middle image using the “adjust levels” command; lower panel, enhancement of diameter “1”/diameter “2” using the “line” tool. The original versions of the stored pairs of images are also provided in Additional file 1: Figure S1

During an initial, 60-min CV period, PEEP was to be optimized according to consecutive assessments of RV function with TEE; these TEE assessments were termed as patient safety measurements (SMs). A 7-MHz transducer connected to a Vivid 7 Expert machine (General Electric) was passed into the esophagus; the TEE probe was to be left within the esophagus throughout the study. The first (baseline) TEE-SM comprised a midesophageal, four-chamber view enabling determination of RV end-diastolic area (RVEDA) and left ventricular end-diastolic area (LVEDA). An RVEDA/LVEDA of > 0.8 triggered at least one downward PEEP titration of 2–3 cmH_2_O, with a consequent, equal decrease in CV-mPaw. The pre-HFO CV-mPaw was used for the subsequent setting of HFO mPaw (see below). Consequently, the pre-HFO PEEP reduction(s) were aimed at both immediately improving RV function and minimizing the risk of subsequent RVEDA/LVEDA > 0.90 (i.e., RV failure [9]) due to high-HFO-associated intrathoracic pressures. Any adjustment in pre-HFO CV settings was to be followed by ≥ 15 min of “stabilization” (i.e., CV with settings maintained unchanged). The TEE-SM was to be repeated within 15–20 min after a PEEP decrease (Fig. 1). Failure to achieve RVEDA/LVEDA < 0.9 after two consecutive PEEP reductions was to result in protocol termination. Study physiological measurements, i.e., gas exchange, respiratory mechanics (by rapid end-inspiratory/end-expiratory airway occlusion and determination of the respective plateau pressures [14]), and hemodynamics [14, 15], and TEE midesophageal, four-chamber, and transgastric, two-chamber, short-axis views (to determine the eccentricity index [9]) were obtained within 50–60 min after study initiation (Figs. 1 and 2).

#### HFO initiation—SpO_2_/TEE-guided setting of HFO mPaw—part 2 of optimization phase (Fig. 1)

This 60–80-min period was aimed at titrating HFO mPaw so as to achieve adequate oxygenation without causing any significant deterioration of RV function relative to pre-HFO CV. Patients were switched to the Sensormedics 3100B HFO ventilator. Care was taken to avoid patient disconnection times of > 3 s. HFO frequency was set at 4 Hz, and FiO_2_ and mPaw were initially set at 1.0 and at 10 cmH_2_O above the mPaw of the preceding CV, respectively. Five minutes thereafter, a 40-s-lasting recruitment maneuver (RM—continuous positive airways pressure of 45 cmH_2_O for 40 s with the oscillator piston off [14, 15]) was performed; RMs were to be canceled if the first study TEE-SM (see above) yielded an RVEDA/LVEDA ratio of ≥ 0.70; in addition, RM protocol safety features are detailed in the Additional file 1. Subsequently, HFO was resumed, and a cuff leak of 3–5 cmH_2_O was placed as previously described [14, 15, 20], and over the subsequent 10 min, HFO-FiO_2_ was adjusted to preceding CV-FiO_2_. Target SpO_2_ was ≥ 95%. Whenever SpO_2_ was < 95%, the initial HFO mPaw was decreased by 3 cmH_2_O, and if after 10 min, SpO_2_ was still < 95%, HFO mPaw was increased by 6 cmH_2_O (i.e., to 13 cmH_2_O above the mPaw of the preceding CV). A TEE-SM was then performed to confirm “RV tolerance of HFO (relative to CV)”; this was defined as RVEDA/LVEDA < 0.9 and not > 0.1 above the RVEDA/LVEDA determined during the latest TEE-SM of pre-HFO CV. If the “RV tolerance criterion” was not met, HFO mPaw was to be reduced by 2–3 cmH_2_O and RVEDA/LVEDA was to be re-determined after 15–20 min. Failure to achieve “RV tolerance of HFO” after two consecutive adjustments in HFO mPaw was to result in protocol termination and patient switching to CV. Maximum period duration was 80 min.

#### Lowest acceptable SpO_2_ during optimization phase

The lowest acceptable SpO_2_ for protocol continuation after any protocol-mandated adjustment in CV or HFO ventilator settings was 90%. During CV, any SpO_2_ drop to < 90% was to be treated with FiO_2_ increase by ≥ 0.1, whenever RVEDA/LVDEA exceeded 0.6; otherwise, PEEP was increased by 2–3 cmH_2_O.

#### HFO strategy testing phase (Fig. 1)

Patients were studied during 60-min testing periods of 4-Hz HFO with cuff leak, or 4-Hz HFO-TGI with cuff leak, or 7-Hz HFO without cuff leak. Employed, stable HFO ventilator settings are presented/reported in Fig. 1 and Table 1. Table 1 also displays estimates of HFO-Vt based on previously published data [1, 15]; see the Additional file 1 for additional details. The HFO mPaw setting associated with SpO_2_ ≥ 95% and “RV tolerance of HFO” was termed as “study HFO mPaw,” and was used during 4-Hz and 7-Hz HFO. During 4-Hz HFO-TGI, mPaw was set at 2–3 cmH_2_O below “study HFO mPaw” to counterbalance a previously determined, TGI-associated increase of similar magnitude in tracheal pressure [14, 15].

**Table 1:** Tested strategies of high-frequency oscillation (HFO)

HFO strategy	4-Hz HFO	4-Hz HFO-TGI	7-Hz HFO
mPaw (cmH_2_O)^a^	+ 10 cmH_2_O^b^	+ 7–8 cmH_2_O^b^	+ 10 cmH_2_O^b^
FiO_2_	FiO_2_ of preceding CV^c^	FiO_2_ of preceding CV^c^	FiO_2_ of preceding CV^c^
ΔP (cmH_2_O)^d^	80–90	80–90	80–90
Bias flow (L/min)	60	60	60
I/E ratio	1/2	1/2	1/2
Cuff leak (cmH_2_O)	3–5	3–5	NA
TGI (L/min)^e^	NA	50% of MV of preceding CV	NA
Estimated Vt (mL)^f^	181.2 ± 6.5	190.0 ± 6.5	118.5 ± 4.1
RM^g^	CPAP of 45 cmH_2_O for 40 s		

*mPaw* mean airway pressure, *TGI* tracheal gas insufflation, *FiO*_*2*_ inspired oxygen fraction, *CV* conventional ventilation, *ΔP* oscillatory pressure amplitude, *I/E ratio* inspiratory-to-expiratory time ratio, *TGI* tracheal gas insufflation, *MV* minute ventilation, *Vt* tidal volume, *RM* recruitment maneuver, *CPAP* continuous positive airway pressure, *NA* not applicable

1 cmH_2_O = 0.098 kPa

^a^Values correspond to the initial setting of the HFO mPaw and are referred to the mPaw of the pre-HFO CV (see also text and Fig. 1)

^b^During HFO-TGI, the mPaw was set at 2–3 cmH_2_O lower than the mPaw of standard HFO to counterbalance the estimated, TGI-induced increase in tracheal pressure [15]; the maximum allowable upper limit of HFO mPaw was 40 cmH_2_O

^c^Provided that peripheral oxygen saturation could be maintained above 90%

^d^Corresponds to actual ventilator-displayed values after the setting of the “Power” within 80–90% of its maximal value

^e^TGI FiO_2_ was equal to the FiO_2_ of the preceding CV; see also Supplement to Methods in Additional file 1

^f^Values are mean ± SD; estimates were based on previously published data on Vt delivery during HFO [1], and a previously published Vt estimate of ~ 200 mL for a specific combination of HFO frequency (i.e., 3.5 Hz), *ΔP* (i.e., 90 cmH_2_O), bias flow (i.e., 40 L/min), tracheal tube internal diameter (i.e., 8.5 mm), mPaw level (i.e., 30 cmH_2_O), and respiratory compliance (i.e., ~ 31 cmH_2_O) [15]; further details (including a calculated possible bias and other limitations of these estimates) are reported in Additional file 1

^g^Each HFO strategy was to be preceded by an RM, provided that RM abort criteria were not met; see also text, Fig. 1, and Additional file 1

HFO strategies were employed in random order (Fig. 1 and Additional file 1: Figure S2). “Low-frequency” and “high-frequency” strategy settings approximated those of prior [14] and recent [7, 8] trials, respectively; the feasibility of “high-frequency” (i.e., > 6 Hz) HFO has been previously documented [21]. Within 25–30 min after the start of 7-Hz HFO, the last TEE-SM was performed; an RVEDA/LVEDA of > 0.9 was to trigger completion of the protocol’s physiological measurements over the next 10 min and patient switching to the next ventilatory strategy (Fig. 1); over this 10-min period, oscillatory pressure amplitude was to be (temporarily) increased to 100 cmH_2_O to prevent further PaCO_2_ rise and associated RV stress.

#### Return to CV—concluding phase (Fig. 1)

Following completion of HFO strategy testing, patients were switched to CV; care was taken to avoid patient disconnection times of > 3 s. CV ventilator settings were identical to those of pre-HFO CV after the final PEEP adjustment. Physiological measurements were repeated within 50–60 min of return to CV, and the study protocol was concluded.

### Study TEE measurements

All TEE imaging was performed by a single, experienced echocardiographer [22]. In contrast to TEE-SMs, which were used for the optimization of PEEP and HFO mPaw according to RVEDA/LVEDA, the study TEE measurements were performed “offline” as described below and further detailed in the Additional file 1. The corresponding, study TEE data were collected during the time intervals of the study’s physiological measurements (Fig. 1). The study TEE data were encoded and saved onto Vivid 7 hard disc. Following study completion, another experienced echocardiographer—not aware of the sequence of the tested ventilatory strategies—used (1) the saved TEE midesophageal, four-chamber imaging data to determine the RVEDA, LVEDA, RVEDA/LVEDA, and RV end-systolic area (RVESA); RV fractional area change was then calculated as (RVEDA – RVESA)/RVEDA; tricuspid annular plane systolic excursion (TAPSE) [23] was also determined as a post hoc (unprespecified) outcome and (2) the saved TEE transgastric imaging data to determine the end-diastolic and end-systolic eccentricity index [9, 22].

TEE imaging was focused at determining major RV function variables also used by preceding echocardiographic studies to compare HFO with lung-protective CV [9, 22]. Consequently, we did not assess pulmonary artery pressure or flow by using continuous or pulsed Doppler [9], respectively, or employ Tissue Doppler Imaging to measure myocardial systolic/diastolic velocities.

### Definitions

RV dysfunction: RVEDA/LVEDA > 0.6 to 0.9; RV failure: RVEDA/LVEDA > 0.9; Eccentricity index: quotient of 2 LV diameters plotted as shown in Fig. 2 (lower panel) [9, 22].

### Main outcome measures

The main outcome measures are as follows: *Primary:* RVEDA/LVEDA ratio, and end-diastolic and end-systolic eccentricity index. *Secondary:* PaO_2_/FiO_2_, PaCO_2_, arterial pH, mean intraarterial pressure, cardiac index, and respiratory compliance (during CV).

### Statistical analysis

Additional methodological information is provided in the Additional file 1. According to an a priori power analysis for a repeated measures analysis of variance (ANOVA) design with one, within-subjects, five-level factor (i.e., ventilatory strategy [20]) and a large effect size index of 0.40 [24], 14 patients would be required, for alpha = 0.05 and power = 0.80. To compensate for possible incomplete observations, we pre-specified a sample size of 17 patients. In case of missing of any of the consecutive ventilatory strategy data points, we specified that we would conduct a linear mixed-model analysis instead of ANOVA. In such a case, the analysis would include “ventilatory strategy” as fixed factor and “patient” as random factor. The mixed-model methodology enables efficient use of data from patients with missing values, thus resulting in more precise estimates of the treatment effect [25].

**Fig. 3:**
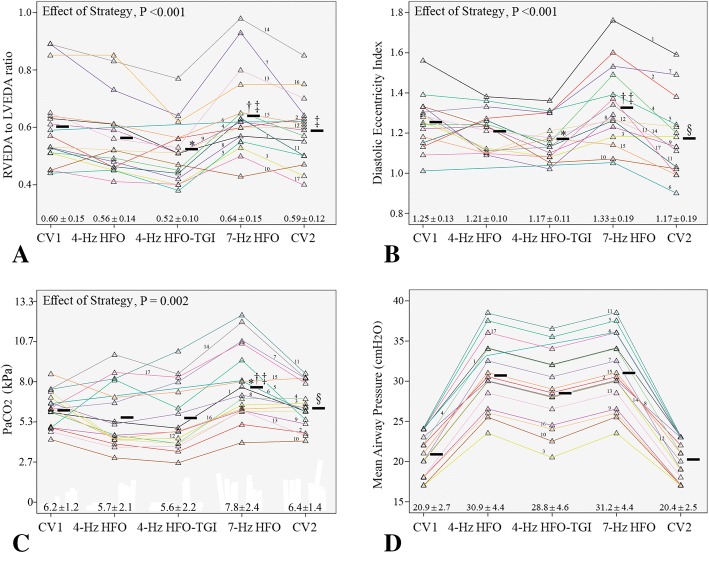
Individual patient data on two primary study outcomes and major determinants of right ventricular function. **a** Primary outcome no. 1, the right-to-left ventricular end-diastolic area ratio (RVEDA/LVEDA). **b** Primary outcome no. 2, the end-diastolic eccentricity index. **c** Determinant of right ventricular function no. 1, the arterial carbon dioxide tension (PaCO_2_). **d** Determinant of right ventricular function no. 2, the mean airway pressure. CV1 first period of conventional ventilation, HFO high-frequency oscillation, TGI tracheal gas insufflation, CV2 second period of conventional ventilation (see also “Methods” and Fig. 1). Numbers (from 1 to 17) just above the colored lines that connect the data points (triangles) indicate patient no.; the color of each “data-point-connecting line” is unique for each one of the patients. Horizontal black bars correspond to mean values. For each ventilatory strategy, summary data are also reported as mean ± SD. “Effect of Strategy” corresponds to the level of significance of the effect of the fixed, within-subjects factor (i.e., ventilatory strategy) in linear mixed-model analysis (see also the “Statistical Analysis” subsection); additional mixed-model data: **a** Percent variation explained 87, calculated as 100 × *R*^2^ value of bivariate linear regression between observed and mixed model-predicted values; **b** percent variation explained, 72; **c** percent variation explained, 74. *p < 0.05 versus CV1. ^†^*p* < 0.05 versus 4-Hz HFO. ^‡^*p* < 0.05 versus 4-Hz HFO-TGI. ^§^*p* < 0.05 versus 7-Hz HFO. Actual *p* values of pairwise comparisons are reported in Additional file 2

Analyses were performed with IBM SPSS Statistics versions 22 and 25. Distribution normality was assessed by Kolmogorov Smirnov test. As further detailed below, there were three patients with missing data points (see also Fig. 3). Consequently, TEE and physiological data collected over the five pre-specified, 10-min intervals (Fig. 1) were analyzed with linear mixed-model analysis; *p* values were adjusted by applying Bonferroni’s correction. Significance was set at two-sided *p* < 0.05.

#### Linear regression

Multiple, stepwise linear regression was used to explore possible associations among ventilatory strategy-associated, relative (i.e., percent) changes in TEE outcome variables (dependent variables) and central venous pressure, RV fractional area change, and PaCO_2_ (independent variables). The first two independent variables were selected because they reflect RV filling pressure and contractility, and PaCO_2_ was selected because it is considered as a major determinant of RV afterload. In addition, we used bivariate linear regression to explore possible associations between the (1) change in PaCO_2_ from baseline CV to 7-Hz HFO (dependent variable) and total respiratory system resistance (Rtot,rs) and (2) the aforementioned PaCO_2_ change and the baseline CV’s change in end-expiratory lung volume due to intrinsic PEEP. Further details are provided in Additional file 1.

## Results

Additional file 1: Figure S2 displays the study flow chart. From February 11, 2014, to October 27, 2016, 17 out of 45 screened ARDS patients (37.8%) were enrolled. Table 2 displays the summary baseline data recorded at 60 min before study initiation; patient-level data on baseline respiratory pathology and mechanics are reported in Additional file 1: Table S3.

**Table 2:** Baseline patient data

Age (years)	60.6 ± 15.2
Sex (male/female), *n*	13/4
Body mass index (kg/m^2^)	24.5 ± 1.9
Predicted body weight (kg)	69.8 ± 7.2
Simplified Acute Physiology Score II^a^	42.9 ± 11.2
Murray score^a^	3.4 ± 0.3
Tracheal tube ID (for male/female patient) (mm)^a^	8.5/8.0
Ventilator mode	Volume assist-control
Inspiratory-to-expiratory time ratio^a^	1/2
FiO_2_ (%)^a^	61.2 ± 9.4
PEEPe (cmH_2_O)^a^	14.7 ± 1.9
Tidal volume (L)/(mL/kg predicted body weight)^a^	0.44 ± 0.46/6.3 ± 0.4
Square-wave inspiratory flow (L/s)^a^	0.67 ± 0.07
Peak airway pressure (cmH_2_O)^a^	43.0 ± 6.2
Plateau airway pressure (cmH_2_O)^a^	30.4 ± 4.4
Mean airway pressure (cmH_2_O)^a^	21.6 ± 2.7
PEEPi (cmH_2_O)^a^	1.0 ± 0.6
Driving pressure (cmH_2_O)^b^	14.7 ± 4.0
C,rs (mL/cmH_2_O)^b^	32.4 ± 10.1
Rtot,rs (cmH_2_O/L.s)^b^	18.5 ± 4.8
ΔEELV (mL)^b^	31.0 ± 19.8
SpO_2_ (%)	91.2 ± 1.9
PaO_2_/FiO_2_	108.4 ± 15.0
PaCO_2_ (kPa)	6.5 ± 1.5
Arterial pH	7.35 ± 0.10
Oxygenation index	20.5 ± 5.0
Time from ARDS diagnosis to study enrollment (h)	42.0 ± 11.2
ARDS etiology	
Hospital-acquired pneumonia, *n* (%)	11 (64.7)
Community-acquired pneumonia, *n* (%)	2 (11.8)
Intra-abdominal sepsis, *n* (%)	2 (11.8)
Other, *n* (%)^c^	2 (11.8)

Values are mean ± SD unless otherwise specified. For males, predicted body weight was calculated as 50 + [height (cm) − 152.4] × 0.91; for females as 45.5 + [height (cm) − 152.4] × 0.91

*ID* internal diameter, *FiO*_*2*_ inspired oxygen fraction, *PEEPe* external positive end-expiratory pressure, *PEEPi* intrinsic positive end-expiratory pressure, *C,rs* respiratory system compliance, *Rtot,rs* respiratory system resistance *ΔEELV* chang e in end-expiratory lung volume due to PEEPi, *PaO*_*2*_ arterial oxygen tension, *SpO*_*2*_ peripheral oxygen saturation, *PaCO*_*2*_ arterial carbon dioxide tension, *ARDS* acute respiratory distress syndrome, *MOF* multiple organ failure

^a^Variables determined/recorded within 1 h before study enrolment

^b^Variables calculated according to standard formulas presented in Additional file 1

^c^Includes one case of bilateral pulmonary contusions and one case of massive blood transfusion

Recruitment maneuvers were canceled or aborted in five patients (see also Additional file 1: Methods). Seven-hertz HFO was discontinued at 40 min in two patients (see Fig. 1 and Additional file 1). However, the criteria for early protocol termination (Fig. 1) were not met in any case. Additional details regarding the setting of study HFO mPaw, patient safety, the stable hemodynamic support (including vasopressor infusion rates) throughout the study period, and in-hospital outcome are provided in Additional file 1.

### Missing data

Patient no. 6 did not receive the 60-min sessions of 4-Hz HFO and 4-Hz HFO-TGI because of HFO ventilator malfunction and return to CV just after the completion of 60 min of 7-Hz HFO. In patient no. 8, the concluding TEE measurements of CV (Fig. 1) were not performed because the echocardiographer was asked to emergently assist in the resuscitation of a young trauma patient in cardiac arrest. Lastly, in patient no. 16, the protocol’s transgastric TEE views were not saved on the Vivid 7 machine’s hard disc due to operator error, and the eccentricity index could not be determined. Consequently, for each one of the five time intervals of physiologic measurements (Fig. 1), the study data were available from at least 15 patients.

### Effect of ventilatory strategy on RV function

New-onset RV dysfunction (*n* = 5) or RV failure (*n* = 2) developed in a total of 7 out of 17 patients (41.2%) during 7-Hz HFO. In all cases, this “new RV dysfunction or failure” was effectively reversed (i.e., RVEDA/LVEDA dropped below 0.60 or 0.90, respectively) following discontinuation of 7-Hz HFO and transition to another ventilatory strategy, i.e., 4-Hz HFO (*n* = 2), or 4-Hz HFO-TGI (*n* = 4), or post-HFO CV (*n* = 1). Accordingly, and regarding the total study group, RVEDA/LVEDA was significantly higher during 7-Hz HFO compared to 4-Hz HFO and 4-Hz HFO-TGI; notably, RVEDA/LVEDA was lower during HFO-TGI relative to pre-HFO and post-HFO CV (Fig. 3a).

Similarly to RVEDA/LVEDA, end-diastolic (Fig. 3b) and end-systolic (Table 3) eccentricity indexes increased during 7-Hz HFO relative to 4-Hz HFO, 4-Hz HFO-TGI, and post-HFO CV, and these changes were reversed following discontinuation of 7-Hz HFO and transition to another ventilatory strategy. Notably, end-diastolic eccentricity index was lower during 4-Hz HFO-TGI, and end-systolic eccentricity index was lower during both 4-Hz HFO and 4-Hz HFO-TGI relative to pre-HFO CV (Fig. 3b and Table 3).

**Table 3:** Results of physiological measurements (see also Fig. 3)

Variable	CV1	4 Hz HFO	4 Hz HFO-TGI	7 Hz HFO	CV2	Strategy Effect–*p* value	*R*^2^/(%) of variation explained
ES-eccentricity index	*1.38 ± 0.21*	*1.26 ± 0.10* ^*a*^	*1.17 ± 0.13* ^*a*^	*1.42 ± 0.17* ^*b,c*^	*1.27 ± 0.19* ^*d*^	*< 0.001*	*0.77/(77%)*
RVEDA (cm^2^)	14.9 ± 5.7	13.0 ± 4.3	12.4 ± 3.8	14.8 ± 5.3^c^	13.6 ± 4.7	0.003	0.92/(92%)
LVEDA (cm^2^)	24.8 ± 7.7	23.4 ± 6.7	24.0 ± 6.6	23.1 ± 7.5	23.1 ± 6.6	0.22	0.91 (91%)
FAC of the RV	0.41 ± 0.08	0.41 ± 0.07	0.44 ± 0.07^b^	0.35 ± 0.09^a,b,c^	0.40 ± 0.09^c^	< 0.001	0.78/(78%)
TAPSE (cm)	1.97 ± 0.55	1.88 ± 0.47	2.04 ± 0.55^b^	1.66 ± 0.48^a,b,c^	1.96 ± 0.58^d^	< 0.001	0.90/(90%)
PaO_2_/FiO_2_	139.1 ± 20.5	249.6 ± 53.6^a^	246.4 ± 77.6^a^	248.1 ± 78.0^a^	155.0 ± 48.2^b,c,d^	< 0.001	0.53/(53%)
ScvO_2_ (%)	68.4 ± 4.7	74.4 ± 5.1^a^	74.3 ± 5.1^a^	74.2 ± 5.8^a^	70.6 ± 4.2^b,c,d^	< 0.001	0.74 (74%)
Arterial pH	7.39 ± 0.09	7.42 ± 0.14	7.43 ± 0.14	7.29 ± 0.12^a,b,c^	7.36 ± 0.09^d^	< 0.001	0.75/(75%)
Shunt fraction	0.34 ± 0.07	0.21 ± 0.07^a^	0.23 ± 0.08^a^	0.23 ± 0.10^a^	0.35 ± 0.10^b,c,d^	< 0.001	0.66/(66%)
Oxygenation Index	16.1 ± 5.4	13.1 ± 4.2^a^	12.7 ± 4.0^a^	14.4 ± 6.8	14.1 ± 4.0	0.03	0.66/(66%)
End-insp. Pplateau (cmH_2_O)^e^	29.9 ± 4.5				27.9 ± 4.5^a^		
End-exp. Pplateau (cmH_2_O)^e^	16.4 ± 2.5				15.9 ± 2.3^f^		
Driving pressure (cmH_2_O)^e^	13.5 ± 3.7				12.0 ± 4.1^a^		
C,rs (mL/cmH_2_O)^e^	35.1 ± 11.5				41.6 ± 17.9^a^		
MAP (kPa)	10.9 ± 0.9	12.3 ± 1.4^a^	12.0 ± 1.2^a^	11.5 ± 1.2	11.4 ± 0.7	0.01	0.62/(62%)
Heart rate (bpm)	89.1 ± 18.8	90.0 ± 18.7	90.0 ± 19.0	99.1 ± 23.7^b,c^	92.2 ± 21.0	0.002	0.88 (88%)
CVP (kPa)	1.7 ± 0.6	1.8 ± 0.5	1.8 ± 0.5	1.9 ± 0.5	1.7 ± 0.5	0.86^g^	
Cardiac index (L/min/m^2^)	3.25 ± 0.38	3.35 ± 0.29	3.43 ± 0.36	3.11 ± 0.40^>c^	3.33 ± 0.34	0.009	0.61 (61%)
Stroke volume index (mL/m^2^)	38.3 ± 10.0	39.2 ± 10.8	40.4 ± 12.2	33.5 ± 10.1^a,b,c^	38.2 ± 10.3^d^	0.001	0.90 (90%)
DO_2_I (mL/min/m^2^)	363.6 ± 64.6	401.6 ± 525^a^	407.5 ± 49.6^a^	368.5 ± 74.7^,c^	373.1 ± 59.1^b,c^	< 0.001	0.83 (83%)
VO_2_I (mL/min/m^2^)	103.8 ± 28.5	109.3 ± 26.7	109.3 ± 28.0	98.9 ± 34.6	99.2 ± 30.6	0.11	0.70 (70%)
SVRI (dynes/cm^5^/s/m^2^)^/^	1744 ± 408	1911 ± 373	1820 ± 364	1905 ± 370	1783 ± 322	0.07	0.82 (82%)

Values are mean ± SD. *CV1* first protocol period of conventional ventilation, *HFO* high-frequency oscillation, *TGI* tracheal gas insufflation, *CV2* second protocol period of conventional ventilation (see also Fig. 1); “Strategy effect—*p* value” pertains to the level of significance of the fixed factor “ventilatory strategy” as determined by linear mixed model analysis (see also “Statistical Analysis” of the current text and Additional file 1); “*R*^2^/(%) variation explained” has been derived by bivariate linear regression between observed and mixed model-predicted values (see also legend of Fig. 3); *ES* end-systolic, *RVEDA* right-ventricular end-diastolic area, *LVEDA* left ventricular end-diastolic area, *RV* right ventricle, *FAC of the RV* RV fractional area change between end-diastole and end-systole [calculated as RVEDA-to-RV end-systolic area difference divided by RVEDA), *TAPSE* tricuspid annular plane systolic excursion, *PaO*_*2*_ arterial oxygen tension, *FiO*_*2*_ inspiratory oxygen fraction, *ScvO*_*2*_ central venous oxygen saturation, *End-insp.* end-inspiratory, *End-exp.* end-expiratory, *Pplateau* plateau pressure, *C*,*rs* quasistatic compliance of the respiratory system, *MAP* mean arterial pressure, *bpm* beats per min, *CVP* central venous pressure, *DO*_*2*_*I* oxygen delivery index, *VO*_*2*_*I* oxygen consumption index, *SVRI* systemic vascular resistance index. Primary outcome (i.e., ES-eccentricity index) data are highlighted in italics

^a^*p* < 0.05 versus CV1

^b^*p* < 0.05 versus 4 Hz HFO

^c^*p* < 0.05 versus 4 Hz HFO-TGI

^d^*p* < 0.05 versus 7 Hz HFO

^e^Variables compared between CV1 and CV2 by a paired t-test. Actual *p* values of pairwise comparisons are reported in Additional file 2

^f^In one patient, CV1 external positive end-expiratory pressure (PEEP) was 18 cmH_2_O and was set at 15 cmH_2_O during CV2 by error of the attending investigator (see also Fig. 1)

^g^*p* value determined by one-way analysis of variance (factor = ventilatory strategy) because convergence was not achieved during the “MIXED procedure” and the validity of mixed-model fit was uncertain

Other TEE results were also suggestive of a reversible deterioration of RV function during 7-Hz HFO. More specifically, RVEDA was higher during 7-Hz HFO compared to 4-Hz HFO-TGI, whereas LVEDA was not significantly affected by ventilatory strategy. Fractional area change of the RV [calculated as (RVEDA – RV end-systolic area)/RVEDA] and TAPSE was lower during 7-Hz HFO compared to 4-Hz HFO and 4-Hz HFO-TGI and pre/post-HFO CV. Lastly, fractional area change of the RV was higher during 4-Hz HFO-TGI compared to 4-Hz HFO, 7-Hz HFO, and post-HFO CV, whereas TAPSE was higher during 4-Hz HFO-TGI compared to 4-Hz HFO (Table 3).

### Gas exchange

Oxygenation and shunt fraction improved during HFO strategies compared to pre-HFO CV; these changes were either largely or fully reversed during post-HFO CV (Table 3). PaO_2_/FiO_2_ exceeded 150 [12, 13] in 14/17 (76.5%), and 16/16 (100.0%) and 15/16 (93.8%) patients during 7-Hz HFO, 4-Hz HFO and 4-Hz HFO-TGI, respectively. Oxygenation index was lower during 4-Hz HFO and 4-Hz HFO-TGI compared to pre-HFO CV (Table 3).

PaCO_2_ increased and arterial pH decreased during 7-Hz HFO compared to all other ventilatory strategies; the 7-Hz HFO-induced worsening in PaCO_2_ (Fig. 3c) and pH (Table 3) was effectively reversed after its discontinuation and transition to another strategy. While on 7-Hz HFO, 10/17 patients (58.8%) exhibited “high PaCO_2_s” of 6.4 to 12.4 kPa [12, 13], while being ventilated at “high-mPaws” of 30.0 to 38.5 cmH_2_O [9] (Fig. 3c, d); 5 of these patients were among those who developed “new RV dysfunction (*n* = 3) or failure (*n* = 2).”

### Respiratory mechanics

Respiratory compliance, end-inspiratory plateau pressure, and driving pressure of post-HFO CV were improved compared to pre-HFO CV (Table 3).

### Hemodynamics

The consecutive use of different ventilatory strategies was associated with relatively minor hemodynamic alterations. Mean arterial pressure was higher (by approximately 1.1–1.3 kPa on average) during 4-Hz HFO and 4-Hz HFO-TGI compared to pre-HFO CV. Cardiac index was relatively stable throughout the study period, but it was lower (by approximately 10% on average) during 7-Hz HFO relative to 4-Hz HFO-TGI. Mean heart rate was 10% higher during 7-Hz HFO compared to 4-Hz HFO and 4-Hz HFO-TGI. Stroke volume index was lower during 7-Hz HFO compared to all other ventilatory strategies. Central venous pressure and oxygen consumption index were not significantly affected by ventilatory strategy. Oxygen delivery index was lower during 7-Hz HFO and pre/post-HFO CV compared to 4-Hz HFO-TGI, and during pre/post-HFO CV compared to 4-Hz HFO. Systemic vascular resistance index did not exhibit any significant change during the study period (Table 3).

### Linear regression

See Additional file 1 for details. Percent changes in PaCO_2_ among tested ventilatory strategies were predictive of concurrent percent changes in measures of RV function (*R*^2^ = 0.21–0.43; Additional file 1: Figure S3). Baseline CV Rtot,rs was predictive of the percent change in PaCO_2_ between baseline CV and 7-Hz HFO (*R*^2^ = 0.27, *p* = 0.03) (Additional file 1: Figure S4).

## Discussion

In this physiological study of 17 moderate-to-severe ARDS patients, a short period of ≤ 60 min of 7 Hz HFO without cuff leak was associated with acute, reversible worsening of RV function compared to 4-Hz HFO with cuff leak and with/without TGI, and CV. Seven-Hz HFO was also associated with CO_2_ retention and acidosis, likely contributing to RV dysfunction. The tested 7-Hz HFO strategy was aimed at approximating the HFO ventilatory conditions of the recent, large, and neutral/negative HFO trials [7, 8]. These trials actually assessed the effect of HFO on lung protection without concurrently focusing on RV protection [9, 10]; according to our results, simultaneous RV exposure to high intrathoracic pressures and hypercapnia can promptly cause new RV dysfunction or failure.

The tested 4-Hz HFO strategies resembled to our previously employed strategy of intermittent HFO-TGI, interspersed with lung-protective CV [14]. This potentially beneficial HFO strategy improved oxygenation, likely through lung recruitment [5, 14, 15, 20, 26], without concurrent deterioration of PaCO_2_ relative to pre-HFO-TGI CV [14, 15, 20, 26, 27]. The HFO-TGI-induced increase in aerated lung volume was indicated by the lower plateau pressures and higher respiratory compliance of post-HFO-TGI CV versus pre-HFO-TGI CV [14, 28]. Furthermore, in contrast to the OSCILLATE trial [8], in our preceding HFO-TGI trial [14] and physiological studies [15, 18, 20, 26, 27] as well (total number of HFO-treated patients, 137), we could not determine any “low-frequency” HFO strategy-related, hemodynamic deterioration relative to CV. Accordingly, in the present study, 4-Hz HFO strategies resulted in similar hemodynamics and similar or even improved TEE measures of RV function compared to CV.

In the current study, oxygenation exhibited similar improvements of approximately 77–80% during all tested HFO strategies relative to pre-HFO CV, indicating lung recruitment [5, 14, 15, 20, 26]. Furthermore, post-HFO versus pre-HFO respiratory compliance was higher and end-inspiratory plateau and driving pressure were lower at the same administered Vt (Fig. 1 and Table 3), again suggesting an increase in aerated lung volume during post-HFO versus pre-HFO CV [14, 28].

In ARDS, main goals of ventilation include adequate gas exchange, lung protection, and RV protection [12, 17]. Factors increasing the risk of RV failure include pneumonia as ARDS cause PaO_2_/FiO_2_ < 150, driving pressure ≥ 18 cmH_2_O, and PaCO_2_ ≥ 6.4 kPa [12, 13]. In the present study, 2, 3, and 4 risk factors were present at baseline in 7, 6, and 3 patients, respectively. The moderate-to-severe oxygenation disturbance was effectively reversed and mean shunt fraction decreased by ≥ 0.12 during HFO strategies (Table 3), suggesting reversal of regional lung derecruitment [26] and attenuation of hypoxic pulmonary vasoconstriction [29]—both mechanisms of RV unloading [12]. Such mechanisms were likely partly counterbalanced by a high-mPaw-associated pulmonary microvascular closure [12] during all HFO strategies. Notably, this high-mPaw afterloading effect on the RV may be attenuated by the recruitment of dependent lung regions [9, 12, 26], and such a potential mechanism could have contributed to the observed, favorable RV function results of 4-Hz HFO and 4-Hz HFO-TGI versus CV (Fig. 3 and Table 3). During 7-Hz HFO, RV unloading mechanisms were likely overwhelmed by the acute, hypercapnic acidosis-induced, pulmonary vasoconstriction [12, 30–32]. This interpretation is supported by the linear associations between changes in PaCO_2_ and changes in RVEDA/LVEDA, and the eccentricity indexes (Additional file 1: Results and Figure S3); the absolute values of the eccentricity indexes indicated RV pressure overload during 7-Hz HFO (Fig. 3 and Table 3) [9, 33].

Clinical evidence-based RV function concerns regarding HFO pertain to the prolonged (i.e., ≥ 24 h) and combined use of a “high mPaw” (i.e., ≥ 30 cmH_2_O) and a “high frequency” (e.g., 6–7 Hz) resulting in severe hypercapnia (i.e., PaCO_2_ ≥ 6.7 kPa) [8, 34–36]. These factors predispose to acute cor pulmonale [9, 12, 13]. Current “short-term” results are consistent with the hypothesis that RV protection might be achievable during 4-Hz HFO or HFO-TGI with cuff leak contributing to PaCO_2_ control [36].

### Limitations

Study sample size was relatively small and included ARDS patients with probably higher lung recruitability [37], thereby casting doubt upon results’ generalizability as regards “short-term” “low-frequency” HFO/HFO-TGI. However, higher recruitability is more frequent among patients with poorer oxygenation [37]. Therefore, further study of “low-frequency” HFO/HFO-TGI seems meaningful, especially in patients with PaO_2_/FiO_2_ < 100 hypoxemia [38].

We did not directly measure tracheal pressure to titrate the mPaw of tested HFO strategies to an “identical” mean tracheal pressure level [15, 20]. Notably, prior measurements of tracheal pressure during “low-frequency” HFO/HFO-TGI indicated mean pressure drops of 5–7 cmH_2_O along endotracheal tubes with cuff leak and internal diameters of 7.5–9.0 mm [14, 15]. This indicates only partial transmission of set HFO mPaws to the lung parenchyma. Pressure drops along 7.5–9.0-mm endotracheal tubes have not been reported to-date for 7-Hz HFO without a cuff leak. Consequently, our RV function results could be partly due to unmeasured differences in tracheal and alveolar pressures between “low-frequency” and “high-frequency” HFO strategies. Moreover, we did not test a 7-Hz HFO strategy with cuff leak. However, our objective was to “approximately reproduce” HFO ventilatory conditions of unfavorable [7, 8] and favorable [14] HFO studies and compare their “short-term” effect on the RV. Τhis “simulation” was feasible only according to standard HFO settings (i.e., mPaw, frequency, and cuff leak) reported by all the aforementioned studies.

In addition, we did not systematically record PICCO *plus*-derived extravascular lung water and pulmonary vascular permeability; however, it was unlikely to detect significant changes in such variables in a short-lasting physiological study; indeed, longer lasting prior studies have reported either slightly favorable or neutral results for HFO strategies versus CV [18, 22].

Lastly, we did not use transthoracic Doppler echocardiography to assess systolic pulmonary artery pressure (SPAP) by measuring tricuspid regurgitation peak velocity (TVR) [39]. However, TVR measurements would have increased protocol complexity since they require obtainment of the best alignment between regurgitant flow and Doppler signal [39]; indeed, the repeatability of this condition might have proven challenging during different ventilatory techniques and a potentially changing extent of lung recruitment and inflation that might even modify the position of the heart. Furthermore, RV dysfunction was absent (i.e., RVEDA/LVEDA was < 0.6) on several measurements’ time-points (Fig. 3), and this might have been associated with “trivial” regurgitant jets [39] and possible underestimation of SPAP. Consequently, the comparability of TVR-estimated SPAP among the tested ventilatory strategies might have been uncertain.

### Clinical and research implications

Our “short-term” results imply that in moderate-to-severe ARDS with favorable oxygenation response to HFO, “low-frequency” HFO strategies with cuff leak may augment lung recruitment without worsening RV function. Future research should evaluate the long-term use of such HFO strategies (with mPaw “optimization” as described in “Methods”), in severe ARDS [19, 38]. TGI limitations have been detailed elsewhere [15]. In our current routine practice, we consider the intermittent [14, 27] use of “low-frequency” HFO-TGI with cuff leak {if tracheal tube internal diameter (ID) ≥ 8.0 mm [15]} or “low-frequency” HFO with cuff leak (if tracheal tube ID = 7.0–7.5 mm [15]) in ARDS patients fulfilling the following criteria: PaO_2_/FiO_2_ < 150 [14, 19, 37, 38], while on low-Vt CV with FiO_2_ ≥ 60%, PEEP ≥ 14 cmH_2_O [37], and driving pressure ≥ 15 cmH_2_O [40]. Sixteen out of 17 (94%) of the current study’s participants fulfilled ≥ 2 of the aforementioned criteria.

## Conclusions

In moderate-to-severe ARDS exhibiting favorable oxygenation response to HFO with mPaw “optimized” according to pre-specified SpO_2_ and TEE-derived RVEDA/LVEDA criteria, short-term 4-Hz HFO or 4-Hz HFO-TGI with cuff leak resulted in better RV function compared to 7-Hz HFO without cuff leak, and similar or even improved RV function relative to lung protective CV. These results could be partly explained by an effective PaCO_2_ control during 4-Hz HFO and 4-Hz HFO-TGI.

## Additional files

Additional file 1: Supplemental Methods and Results. (DOC 2 kb)

Additional file 2: Actual *p* values for results reported in Fig. 3 of the main paper. (RTF 2267 kb)

## Abbreviations

ANOVA: Analysis of variance
ARDS: Acute respiratory distress syndrome
CV: Conventional ventilation
FiO_2_: Inspiratory O_2_ fraction
HFO: High-frequency oscillation
LVEDA: Left ventricular end-diastolic area
mPaw: Mean airway pressure
PEEP: Positive end-expiratory pressure
PiCCO: Pulse-induced contour cardiac output
RCT: Randomized clinical trial
Rtot,rs: Total respiratory system resistance
RV: Right ventricular
RVEDA: RV end-diastolic area
SM: Safety measurement
SPAP: Systolic pulmonary artery pressure
SpO_2_: Peripheral oxygen saturation
TAPSE: Tricuspid annular plane systolic excursion
TEE: Transesophageal echocardiography
TGI: Tracheal gas insufflation
TRV: Tricuspid regurgitation peak velocity
Vt: Tidal volume

## References

[1] Hager DN, Fessler HE, Kaczka DW, Shanholtz CB (2007). Tidal volume delivery during high-frequency oscillatory ventilation in adults with acute respiratory distress syndrome. Crit Care Med.

[2] Fort P, Farmer C, Westerman J (1997). High-frequency oscillatory ventilation for adult respiratory distress syndrome—a pilot study. Crit Care Med.

[3] Mehta S, Lapinsky SE, Hallett DC (2001). Prospective trial of high-frequency oscillation in adults with acute respiratory distress syndrome. Crit Care Med.

[4] Derdak S, Mehta S, Stewart TE, Multicenter Oscillatory Ventilation For Acute Respiratory Distress Syndrome Trial (MOAT) Study Investigators (2002). High-frequency oscillatory ventilation for acute respiratory distress syndrome in adults: a randomized, controlled trial. Am J Respir Crit Care Med.

[5] Ferguson ND, Chiche JD, Kacmarek RM (2005). Combining high-frequency oscillatory ventilation and recruitment maneuvers in adults with early acute respiratory distress syndrome: the Treatment with Oscillation and an Open Lung Strategy (TOOLS) trial pilot study. Crit Care Med.

[6] Sud S, Sud M, Friedrich JO (2010). High frequency oscillation in patients with acute lung injury and acute respiratory distress syndrome (ARDS): systematic review and meta-analysis. BMJ.

[7] Young D, Lamb SE, Shah S, For the OSCAR Study Group (2013). High-frequency oscillation for acute respiratory distress syndrome. N Engl J Med.

[8] Ferguson ND, Cook DJ, Guyatt GH, OSCILLATE Trial Investigators, Canadian Critical Care Trials Group (2013). High-frequency oscillation in early acute respiratory distress syndrome. N Engl J Med..

[9] Guervilly C, Forel JM, Hraiech S (2012). Right ventricular function during high-frequency oscillatory ventilation in adults with acute respiratory distress syndrome. Crit Care Med.

[10] Guervilly C, Roch A, Papazian L (2013). High-frequency oscillation for ARDS. N Engl J Med.

[11] Vieillard-Baron A, Price LC, Matthay MA (2013). Acute cor pulmonale in ARDS. Intensive Care Med.

[12] Vieillard-Baron A, Matthay M, Teboul JL (2016). Experts’ opinion on management of hemodynamics in ARDS patients: focus on the effects of mechanical ventilation. Intensive Care Med.

[13] Mekontso Dessap A, Boissier F, Charron C (2016). Acute cor pulmonale during protective ventilation for acute respiratory distress syndrome: prevalence, predictors, and clinical impact. Intensive Care Med.

[14] Mentzelopoulos SD, Malachias S, Zintzaras E (2012). Intermittent recruitment with high-frequency oscillation/tracheal gas insufflation in acute respiratory distress syndrome. Eur Respir J.

[15] Mentzelopoulos SD, Malachias S, Kokkoris S, Roussos C, Zakynthinos SG (2010). Comparison of high-frequency oscillation and tracheal gas insufflation versus standard high-frequency oscillation at two levels of tracheal pressure. Intensive Care Med.

[16] Ranieri VM, Suter PM, Tortorella C, et al. Effect of mechanical ventilation on inflammatory mediators in patients with acute respiratory distress syndrome: a randomized controlled trial. JAMA 1999;282(1):54–61. 10.1001/jama.282.1.54 10404912

[17] Network ARDS, Brower RG, Matthay MA, Morris A, Schoenfeld D, Thompson BT, Wheeler A (2000). Ventilation with lower tidal volumes as compared with traditional tidal volumes for acute lung injury and the acute respiratory distress syndrome. N Engl J Med.

[18] Vrettou CS, Zakynthinos SG, Malachias S, Mentzelopoulos SD (2014). The effect of high-frequency oscillatory ventilation combined with tracheal gas insufflation on extravascular lung water in patients with acute respiratory distress syndrome: a randomized, crossover, physiologic study. J Crit Care.

[19] Definition Task Force ARDS, Ranieri VM, Rubenfeld GD, Thompson BT (2012). Acute respiratory distress syndrome: the Berlin definition. JAMA.

[20] Mentzelopoulos SD, Roussos C, Koutsoukou A (2007). Acute effects of combined high-frequency oscillation and tracheal gas insufflation in severe acute respiratory distress syndrome. Crit Care Med.

[21] Fessler HE, Hager DN, Brower RG. Feasibility of very high-frequency ventilation in adults with acute respiratory distress syndrome. Crit Care Med 2008;36(4):1043–1048. 10.1097/01.CCM.0b013e318168fcab 18379227

[22] Ursulet L, Roussiaux A, Belcour D, et al. Right over left ventricular end-diastolic area relevance to predict hemodynamic intolerance of high-frequency oscillatory ventilation in patients with severe ARDS. Ann Intensive Care 2015;5(1):25. 10.1186/s13613-015-0068-6 PMC4573736 26380993

[23] Schmid E, Hilberath JN, Blumenstock G (2015). Tricuspid annular plane systolic excursion (TAPSE) predicts poor outcome in patients undergoing acute pulmonary embolectomy. Heart Lung Vessel.

[24] Cohen J. The analysis of variance and covariance. In: Cohen J, ed. Statistical power analysis for the behavioral sciences. 2nd Ed. Lawrence Erlbaum Associates, Hillsdale, 1988; pp. 273–406.

[25] Brown H, Prescott R, Brown H, Prescott R (2006). Introduction. Applied mixed models in medicine.

[26] Mentzelopoulos SD, Theodoridou M, Malachias S (2011). Scanographic comparison of high frequency oscillation with versus without tracheal gas insufflation in acute respiratory distress syndrome. Intensive Care Med.

[27] Vrettou CS, Zakynthinos SG, Malachias S, Mentzelopoulos SD (2013). High-frequency oscillation and tracheal gas insufflation in patients with severe acute respiratory distress syndrome and traumatic brain injury: an interventional physiological study. Crit Care.

[28] Henzler D, Pelosi P, Dembinski R (2005). Respiratory compliance but not gas exchange correlates with changes in lung aeration after a recruitment maneuver: an experimental study in pigs with saline lavage acute lung injury. Crit Care.

[29] Naeije R, Brimioulle S (2001). Physiology in medicine: importance of hypoxic pulmonary vasoconstriction in maintaining arterial oxygenation during acute respiratory failure. Crit Care.

[30] Brimioulle S, Lejeune P, Vachiery JL, Leeman M, Melot C, Naeije R. Effects of acidosis and alkalosis on hypoxic pulmonary vasoconstriction in dogs. Am J Phys 1990; 58(2 Pt 2):H347-H353. 10.1152/ajpheart.1990.258.2.H347 2309902

[31] Puybasset L, Stewart T, Rouby JJ (1994). Inhaled nitric oxide reverses the increase in pulmonary vascular resistance induced by permissive hypercapnia in patients with acute respiratory distress syndrome. Anesthesiology.

[32] Mekontso Dessap A, Charron C, Devaquet J (2009). Impact of acute hypercapnia and augmented positive end-expiratory pressure on right ventricle function in severe acute respiratory distress syndrome. Intensive Care Med.

[33] Ryan T, Petrovic O, Dillon JC, Feigenbaum H, Conley MJ, Armstrong WF (1985). An echocardiographic index for separation of right ventricular volume and pressure overload. J Am Coll Cardiol.

[34] Goffi A, Ferguson ND (2014). High-frequency oscillatory ventilation for early acute respiratory distress syndrome in adults. Curr Opin Crit Care.

[35] Nin N, Muriel A, Peñuelas O, VENTILA Group (2017). Severe hypercapnia and outcome of mechanically ventilated patients with moderate or severe acute respiratory distress syndrome. Intensive Care Med..

[36] Derdak S (2003). High-frequency oscillatory ventilation for acute respiratory distress syndrome in adult patients. Crit Care Med.

[37] Gattinoni L, Caironi P, Cressoni M (2006). Lung recruitment in patients with the acute respiratory distress syndrome. N Engl J Med.

[38] Meade MO, Young D, Hanna S (2017). Severity of hypoxemia and effect of high frequency oscillatory ventilation in ARDS. Am J Respir Crit Care Med.

[39] Bossone E, D’Andrea A, D'Alto M (2013). Echocardiography in pulmonary arterial hypertension: from diagnosis to prognosis. J Am Soc Echocardiogr.

[40] Amato MB, Meade MO, Slutsky AS (2015). Driving pressure and survival in the acute respiratory distress syndrome. N Engl J Med.

