# Supplementary material for: “Low-” versus “high”-frequency oscillation and right ventricular function in ARDS. A randomized crossover study
Source: J Intensive Care. 2018 Sep 4;6:58. doi: 10.1186/s40560-018-0327-3 (PMC6122746; doi:10.1186/s40560-018-0327-3)
Supplement: Supplementary file 2 — Actual p values for results reported in Fig. 3 of the main paper. (RTF 2267 kb) [file 40560_2018_327_MOESM2_ESM.rtf]

ACTUAL P-values for results reported in Figure 3 OF THE MAIN PAPER

Pairwise Comparisons for RVEDA TO LVEDA RATIO a	
(I) Ventilatory Technique	(J) Ventilatory Technique	Mean Difference (I-J)	Std. Error	df	Sig.c	95% Confidence Interval for Differencec	
						Lower Bound	Upper Bound	
CV1	4Hz HFO	.039	.020	14.804	.666	-.026	.103	
	4Hz HFO-TGI	.082*	.016	19.542	.001	.031	.132	
	7 Hz HFO	-.041	.022	18.332	.873	-.112	.031	
	CV2	.020	.018	23.000	1.000	-.036	.076	
4Hz HFO	CV1	-.039	.020	14.804	.666	-.103	.026	
	4Hz HFO-TGI	.043	.016	17.589	.144	-.008	.094	
	7 Hz HFO	-.079*	.023	21.317	.021	-.150	-.009	
	CV2	-.019	.018	22.419	1.000	-.075	.038	
4Hz HFO-TGI	CV1	-.082*	.016	19.542	.001	-.132	-.031	
	4Hz HFO	-.043	.016	17.589	.144	-.094	.008	
	7 Hz HFO	-.122*	.020	19.185	.000	-.184	-.060	
	CV2	-.062*	.014	11.322	.011	-.111	-.013	
7 Hz HFO	CV1	.041	.022	18.332	.873	-.031	.112	
	4Hz HFO	.079*	.023	21.317	.021	.009	.150	
	4Hz HFO-TGI	.122*	.020	19.185	.000	.060	.184	
	CV2	.061	.021	20.807	.094	-.006	.127	
CV2	CV1	-.020	.018	23.000	1.000	-.076	.036	
	4Hz HFO	.019	.018	22.419	1.000	-.038	.075	
	4Hz HFO-TGI	.062*	.014	11.322	.011	.013	.111	
	7 Hz HFO	-.061	.021	20.807	.094	-.127	.006	
Based on estimated marginal means	
*. The mean difference is significant at the .05 level.	
a. Dependent Variable: RVEDA TO LVEDA RATIO.	
c. Adjustment for multiple comparisons: Bonferroni.	


Pairwise Comparisons for End-Diastolic Eccentricity Indexa
	
(I) Ventilatory Technique	(J) Ventilatory Technique	Mean Difference (I-J)	Std. Error	df	Sig.c	95% Confidence Interval for Differencec	
						Lower Bound	Upper Bound	
CV1	4Hz HFO	.057	.031	16.005	.815	-.043	.156	
	4Hz HFO-TGI	.094*	.028	17.664	.038	.003	.185	
	7 Hz HFO	-.074	.040	24.799	.757	-.198	.049	
	CV2	.085	.038	22.946	.371	-.034	.204	
4Hz HFO	CV1	-.057	.031	16.005	.815	-.156	.043	
	4Hz HFO-TGI	.037	.019	15.561	.741	-.026	.101	
	7 Hz HFO	-.131*	.035	19.620	.013	-.241	-.021	
	CV2	.028	.033	18.948	1.000	-.076	.132	
4Hz HFO-TGI	CV1	-.094*	.028	17.664	.038	-.185	-.003	
	4Hz HFO	-.037	.019	15.561	.741	-.101	.026	
	7 Hz HFO	-.168*	.033	14.944	.001	-.277	-.060	
	CV2	-.009	.031	13.527	1.000	-.112	.093	
7 Hz HFO	CV1	.074	.040	24.799	.757	-.049	.198	
	4Hz HFO	.131*	.035	19.620	.013	.021	.241	
	4Hz HFO-TGI	.168*	.033	14.944	.001	.060	.277	
	CV2	.159*	.042	21.139	.010	.028	.290	
CV2	CV1	-.085	.038	22.946	.371	-.204	.034	
	4Hz HFO	-.028	.033	18.948	1.000	-.132	.076	
	4Hz HFO-TGI	.009	.031	13.527	1.000	-.093	.112	
	7 Hz HFO	-.159*	.042	21.139	.010	-.290	-.028	
Based on estimated marginal means	
*. The mean difference is significant at the .05 level.	
a. Dependent Variable: End-Diastolic Eccentricity Index..	
c. Adjustment for multiple comparisons: Bonferroni.	


Pairwise Comparisons for PaCO2a	
(I) Ventilatory Technique	(J) Ventilatory Technique	Mean Difference (I-J)	Std. Error	df	Sig.c	95% Confidence Interval for Differencec	
						Lower Bound	Upper Bound	
CV1	4Hz HFO	4.058	2.961	20.678	1.000	-5.241	13.357	
	4Hz HFO-TGI	4.208	2.666	25.441	1.000	-3.984	12.400	
	7 Hz HFO	-12.188*	3.110	23.892	.006	-21.803	-2.574	
	CV2	-1.688	1.617	9.989	1.000	-7.480	4.103	
4Hz HFO	CV1	-4.058	2.961	20.678	1.000	-13.357	5.241	
	4Hz HFO-TGI	.150	3.305	18.019	1.000	-10.413	10.713	
	7 Hz HFO	-16.246*	3.675	20.749	.002	-27.783	-4.709	
	CV2	-5.746	2.539	13.368	.409	-14.264	2.772	
4Hz HFO-TGI	CV1	-4.208	2.666	25.441	1.000	-12.400	3.984	
	4Hz HFO	-.150	3.305	18.019	1.000	-10.713	10.413	
	7 Hz HFO	-16.396*	3.442	21.989	.001	-27.130	-5.662	
	CV2	-5.896	2.188	14.778	.168	-13.105	1.313	
7 Hz HFO	CV1	12.188*	3.110	23.892	.006	2.574	21.803	
	4Hz HFO	16.246*	3.675	20.749	.002	4.709	27.783	
	4Hz HFO-TGI	16.396*	3.442	21.989	.001	5.662	27.130	
	CV2	10.500*	2.711	15.632	.014	1.650	19.350	
CV2	CV1	1.688	1.617	9.989	1.000	-4.103	7.480	
	4Hz HFO	5.746	2.539	13.368	.409	-2.772	14.264	
	4Hz HFO-TGI	5.896	2.188	14.778	.168	-1.313	13.105	
	7 Hz HFO	-10.500*	2.711	15.632	.014	-19.350	-1.650	
Based on estimated marginal means	
*. The mean difference is significant at the .05 level.	
a. Dependent Variable: PaCO2 (mmHg).	
c. Adjustment for multiple comparisons: Bonferroni.	


ACTUAL P-VALUES FOR RESULTS REPORTED IN TABLE 3 OF THE MAIN PAPER
Pairwise Comparisons for End-Systolic Eccentricity Indexa


(I) Ventilatory Technique
(J) Ventilatory Technique
Mean Difference (I-J)
Std. Error
df
Sig.c
95% Confidence Interval for Differencec


Lower Bound
Upper Bound

CV1
4Hz HFO
.129*
.039
20.885
.037
.005
.252


4Hz HFO-TGI
.221*
.037
18.200
.000
.103
.339


7 Hz HFO
-.045
.041
16.471
1.000
-.176
.086


CV2
.117
.043
17.132
.151
-.022
.256

4Hz HFO
CV1
-.129*
.039
20.885
.037
-.252
-.005


4Hz HFO-TGI
.092
.029
13.762
.069
-.005
.189


7 Hz HFO
-.174*
.034
25.106
.000
-.277
-.070


CV2
-.012
.037
18.036
1.000
-.130
.106

4Hz HFO-TGI
CV1
-.221*
.037
18.200
.000
-.339
-.103


4Hz HFO
-.092
.029
13.762
.069
-.189
.005


7 Hz HFO
-.266*
.031
15.149
.000
-.367
-.164


CV2
-.104
.034
19.378
.069
-.213
.005

7 Hz HFO
CV1
.045
.041
16.471
1.000
-.086
.176


4Hz HFO
.174*
.034
25.106
.000
.070
.277


4Hz HFO-TGI
.266*
.031
15.149
.000
.164
.367


CV2
.162*
.038
17.965
.005
.040
.284

CV2
CV1
-.117
.043
17.132
.151
-.256
.022


4Hz HFO
.012
.037
18.036
1.000
-.106
.130


4Hz HFO-TGI
.104
.034
19.378
.069
-.005
.213


7 Hz HFO
-.162*
.038
17.965
.005
-.284
-.040

Based on estimated marginal means

*. The mean difference is significant at the .05 level.

a. Dependent Variable: End-Systolic Eccentricity Index.

c. Adjustment for multiple comparisons: Bonferroni.


	
	Pairwise Comparisons for RVEDAa
	
(I) Ventilatory Technique	(J) Ventilatory Technique	Mean Difference (I-J)	Std. Error	df	Sig.c	95% Confidence Interval for Differencec	
						Lower Bound	Upper Bound	
CV1	4Hz HFO	1.386	.662	20.388	.491	-.698	3.470	
	4Hz HFO-TGI	1.977	.637	16.963	.065	-.078	4.032	
	7 Hz HFO	.088	.681	22.989	1.000	-2.027	2.203	
	CV2	1.142	.667	22.395	1.000	-.933	3.217	
4Hz HFO	CV1	-1.386	.662	20.388	.491	-3.470	.698	
	4Hz HFO-TGI	.591	.404	18.547	1.000	-.694	1.876	
	7 Hz HFO	-1.298	.476	16.435	.146	-2.839	.243	
	CV2	-.244	.455	19.695	1.000	-1.681	1.194	
4Hz HFO-TGI	CV1	-1.977	.637	16.963	.065	-4.032	.078	
	4Hz HFO	-.591	.404	18.547	1.000	-1.876	.694	
	7 Hz HFO	-1.889*	.440	22.055	.003	-3.262	-.515	
	CV2	-.834	.418	16.736	.623	-2.184	.515	
7 Hz HFO	CV1	-.088	.681	22.989	1.000	-2.203	2.027	
	4Hz HFO	1.298	.476	16.435	.146	-.243	2.839	
	4Hz HFO-TGI	1.889*	.440	22.055	.003	.515	3.262	
	CV2	1.054	.482	16.944	.429	-.499	2.607	
CV2	CV1	-1.142	.667	22.395	1.000	-3.217	.933	
	4Hz HFO	.244	.455	19.695	1.000	-1.194	1.681	
	4Hz HFO-TGI	.834	.418	16.736	.623	-.515	2.184	
	7 Hz HFO	-1.054	.482	16.944	.429	-2.607	.499	
Based on estimated marginal means	
*. The mean difference is significant at the .05 level.	
a. Dependent Variable: RVEDA	
c. Adjustment for multiple comparisons: Bonferroni.	


	
Pairwise Comparisons for LVEDAa
	
(I) Ventilatory Technique	(J) Ventilatory Technique	Mean Difference (I-J)	Std. Error	df	Sig.b	95% Confidence Interval for Differenceb	
						Lower Bound	Upper Bound	
CV1	4Hz HFO	.601	.984	21.103	1.000	-2.484	3.685	
	4Hz HFO-TGI	.026	.972	17.620	1.000	-3.091	3.144	
	7 Hz HFO	1.641	.942	22.116	.956	-1.297	4.578	
	CV2	1.015	.920	20.592	1.000	-1.876	3.905	
4Hz HFO	CV1	-.601	.984	21.103	1.000	-3.685	2.484	
	4Hz HFO-TGI	-.574	.777	21.722	1.000	-3.000	1.851	
	7 Hz HFO	1.040	.746	16.804	1.000	-1.367	3.447	
	CV2	.414	.718	19.170	1.000	-1.862	2.690	
4Hz HFO-TGI	CV1	-.026	.972	17.620	1.000	-3.144	3.091	
	4Hz HFO	.574	.777	21.722	1.000	-1.851	3.000	
	7 Hz HFO	1.614	.730	22.109	.377	-.661	3.890	
	CV2	.988	.701	17.658	1.000	-1.260	3.236	
7 Hz HFO	CV1	-1.641	.942	22.116	.956	-4.578	1.297	
	4Hz HFO	-1.040	.746	16.804	1.000	-3.447	1.367	
	4Hz HFO-TGI	-1.614	.730	22.109	.377	-3.890	.661	
	CV2	-.626	.658	15.067	1.000	-2.788	1.536	
CV2	CV1	-1.015	.920	20.592	1.000	-3.905	1.876	
	4Hz HFO	-.414	.718	19.170	1.000	-2.690	1.862	
	4Hz HFO-TGI	-.988	.701	17.658	1.000	-3.236	1.260	
	7 Hz HFO	.626	.658	15.067	1.000	-1.536	2.788	
Based on estimated marginal means	
a. Dependent Variable: LVEDA.	
b. Adjustment for multiple comparisons: Bonferroni.


	

Pairwise Comparisons for Fractional Area Change of the RVa
	
(I) Ventilatory Technique	(J) Ventilatory Technique	Mean Difference (I-J)	Std. Error	df	Sig.c	95% Confidence Interval for Differencec	
						Lower Bound	Upper Bound	
CV1	4Hz HFO	-.001	.015	18.747	1.000	-.047	.045	
	4Hz HFO-TGI	-.041	.015	22.072	.110	-.086	.005	
	7 Hz HFO	.058*	.018	22.061	.031	.004	.113	
	CV2	.009	.017	22.423	1.000	-.044	.062	
4Hz HFO	CV1	.001	.015	18.747	1.000	-.045	.047	
	4Hz HFO-TGI	-.039*	.011	14.584	.027	-.076	-.003	
	7 Hz HFO	.059*	.015	18.404	.008	.012	.106	
	CV2	.010	.014	21.952	1.000	-.034	.055	
4Hz HFO-TGI	CV1	.041	.015	22.072	.110	-.005	.086	
	4Hz HFO	.039*	.011	14.584	.027	.003	.076	
	7 Hz HFO	.099*	.015	22.403	.000	.053	.145	
	CV2	.050*	.014	16.549	.029	.004	.096	
7 Hz HFO	CV1	-.058*	.018	22.061	.031	-.113	-.004	
	4Hz HFO	-.059*	.015	18.404	.008	-.106	-.012	
	4Hz HFO-TGI	-.099*	.015	22.403	.000	-.145	-.053	
	CV2	-.049	.017	21.661	.096	-.103	.005	
CV2	CV1	-.009	.017	22.423	1.000	-.062	.044	
	4Hz HFO	-.010	.014	21.952	1.000	-.055	.034	
	4Hz HFO-TGI	-.050*	.014	16.549	.029	-.096	-.004	
	7 Hz HFO	.049	.017	21.661	.096	-.005	.103	
Based on estimated marginal means	
*. The mean difference is significant at the .05 level.	
a. Dependent Variable: Fractional Area Change of the RV.	
c. Adjustment for multiple comparisons: Bonferroni.	


Pairwise Comparisons for TAPSEa
	
(I) Ventilatory Technique	(J) Ventilatory Technique	Mean Difference (I-J)	Std. Error	df	Sig.c	95% Confidence Interval for Differencec	
						Lower Bound	Upper Bound	
CV1	4Hz HFO	,042	,058	21,992	1,000	-,138	,222	
	4Hz HFO-TGI	-,126	,056	14,742	,399	-,311	,058	
	7 Hz HFO	,312*	,073	25,440	,002	,089	,536	
	CV2	,019	,076	19,810	1,000	-,221	,259	
4Hz HFO	CV1	-,042	,058	21,992	1,000	-,222	,138	
	4Hz HFO-TGI	-,168*	,040	13,762	,009	-,301	-,035	
	7 Hz HFO	,271*	,062	16,701	,004	,071	,471	
	CV2	-,023	,066	16,660	1,000	-,235	,190	
4Hz HFO-TGI	CV1	,126	,056	14,742	,399	-,058	,311	
	4Hz HFO	,168*	,040	13,762	,009	,035	,301	
	7 Hz HFO	,439*	,060	18,072	,000	,246	,632	
	CV2	,145	,064	16,696	,375	-,063	,353	
7 Hz HFO	CV1	-,312*	,073	25,440	,002	-,536	-,089	
	4Hz HFO	-,271*	,062	16,701	,004	-,471	-,071	
	4Hz HFO-TGI	-,439*	,060	18,072	,000	-,632	-,246	
	CV2	-,293*	,079	24,211	,011	-,538	-,049	
CV2	CV1	-,019	,076	19,810	1,000	-,259	,221	
	4Hz HFO	,023	,066	16,660	1,000	-,190	,235	
	4Hz HFO-TGI	-,145	,064	16,696	,375	-,353	,063	
	7 Hz HFO	,293*	,079	24,211	,011	,049	,538	
Based on estimated marginal means	
*. The mean difference is significant at the .05 level.	
a. Dependent Variable: TAPSE.	
c. Adjustment for multiple comparisons: Bonferroni.	


Pairwise Comparisons for PaO2/FiO2a	
(I) Ventilatory Technique	(J) Ventilatory Technique	Mean Difference (I-J)	Std. Error	df	Sig.c	95% Confidence Interval for Differencec	
						Lower Bound	Upper Bound	
CV1	4Hz HFO	-110.361*	12.440	14.602	.000	-151.426	-69.296	
	4Hz HFO-TGI	-107.161*	18.211	14.820	.000	-167.125	-47.197	
	7 Hz HFO	-108.965*	17.722	15.927	.000	-166.637	-51.292	
	CV2	-15.894	9.956	15.485	1.000	-48.439	16.650	
4Hz HFO	CV1	110.361*	12.440	14.602	.000	69.296	151.426	
	4Hz HFO-TGI	3.200	22.009	24.262	1.000	-64.745	71.145	
	7 Hz HFO	1.396	21.608	26.374	1.000	-64.789	67.581	
	CV2	94.467*	15.874	27.085	.000	45.962	142.972	
4Hz HFO-TGI	CV1	107.161*	18.211	14.820	.000	47.197	167.125	
	4Hz HFO	-3.200	22.009	24.262	1.000	-71.145	64.745	
	7 Hz HFO	-1.804	25.373	29.333	1.000	-78.818	75.211	
	CV2	91.267*	20.709	22.541	.002	26.849	155.685	
7 Hz HFO	CV1	108.965*	17.722	15.927	.000	51.292	166.637	
	4Hz HFO	-1.396	21.608	26.374	1.000	-67.581	64.789	
	4Hz HFO-TGI	1.804	25.373	29.333	1.000	-75.211	78.818	
	CV2	93.071*	20.280	24.553	.001	30.535	155.606	
CV2	CV1	15.894	9.956	15.485	1.000	-16.650	48.439	
	4Hz HFO	-94.467*	15.874	27.085	.000	-142.972	-45.962	
	4Hz HFO-TGI	-91.267*	20.709	22.541	.002	-155.685	-26.849	
	7 Hz HFO	-93.071*	20.280	24.553	.001	-155.606	-30.535	
Based on estimated marginal means	
*. The mean difference is significant at the .05 level.	
a. Dependent Variable: PaO2/FiO2 (mmHg).	
c. Adjustment for multiple comparisons: Bonferroni.	


Pairwise Comparisons for ScvO2a	
(I) Ventilatory Technique	(J) Ventilatory Technique	Mean Difference (I-J)	Std. Error	df	Sig.c	95% Confidence Interval for Differencec	
						Lower Bound	Upper Bound	
CV1	4Hz HFO	-6.179*	1.020	21.569	.000	-9.366	-2.992	
	4Hz HFO-TGI	-5.991*	1.081	23.898	.000	-9.335	-2.648	
	7 Hz HFO	-5.882*	1.303	21.985	.002	-9.946	-1.818	
	CV2	-2.235	1.093	19.114	.549	-5.702	1.232	
4Hz HFO	CV1	6.179*	1.020	21.569	.000	2.992	9.366	
	4Hz HFO-TGI	.188	.858	9.295	1.000	-2.948	3.323	
	7 Hz HFO	.296	1.134	20.855	1.000	-3.263	3.855	
	CV2	3.943*	.885	20.786	.002	1.164	6.723	
4Hz HFO-TGI	CV1	5.991*	1.081	23.898	.000	2.648	9.335	
	4Hz HFO	-.188	.858	9.295	1.000	-3.323	2.948	
	7 Hz HFO	.109	1.190	23.014	1.000	-3.585	3.803	
	CV2	3.756*	.956	23.550	.006	.796	6.716	
7 Hz HFO	CV1	5.882*	1.303	21.985	.002	1.818	9.946	
	4Hz HFO	-.296	1.134	20.855	1.000	-3.855	3.263	
	4Hz HFO-TGI	-.109	1.190	23.014	1.000	-3.803	3.585	
	CV2	3.647	1.201	20.026	.065	-.139	7.433	
CV2	CV1	2.235	1.093	19.114	.549	-1.232	5.702	
	4Hz HFO	-3.943*	.885	20.786	.002	-6.723	-1.164	
	4Hz HFO-TGI	-3.756*	.956	23.550	.006	-6.716	-.796	
	7 Hz HFO	-3.647	1.201	20.026	.065	-7.433	.139	
Based on estimated marginal means	
*. The mean difference is significant at the .05 level.	
a. Dependent Variable: ScvO2.	
c. Adjustment for multiple comparisons: Bonferroni.	


Pairwise Comparisons for Arterial pHa	
(I) Ventilatory Technique	(J) Ventilatory Technique	Mean Difference (I-J)	Std. Error	df	Sig.c	95% Confidence Interval for Differencec	
						Lower Bound	Upper Bound	
CV1	4Hz HFO	-.034	.029	18.019	1.000	-.126	.057	
	4Hz HFO-TGI	-.042	.030	20.065	1.000	-.136	.053	
	7 Hz HFO	.093*	.028	16.223	.041	.003	.183	
	CV2	.023	.033	25.092	1.000	-.079	.125	
4Hz HFO	CV1	.034	.029	18.019	1.000	-.057	.126	
	4Hz HFO-TGI	-.007	.015	16.750	1.000	-.055	.040	
	7 Hz HFO	.127*	.010	11.003	.000	.094	.161	
	CV2	.057	.021	20.279	.117	-.008	.122	
4Hz HFO-TGI	CV1	.042	.030	20.065	1.000	-.053	.136	
	4Hz HFO	.007	.015	16.750	1.000	-.040	.055	
	7 Hz HFO	.135*	.013	16.364	.000	.092	.177	
	CV2	.065	.022	21.993	.086	-.005	.134	
7 Hz HFO	CV1	-.093*	.028	16.223	.041	-.183	-.003	
	4Hz HFO	-.127*	.010	11.003	.000	-.161	-.094	
	4Hz HFO-TGI	-.135*	.013	16.364	.000	-.177	-.092	
	CV2	-.070*	.019	16.383	.023	-.133	-.007	
CV2	CV1	-.023	.033	25.092	1.000	-.125	.079	
	4Hz HFO	-.057	.021	20.279	.117	-.122	.008	
	4Hz HFO-TGI	-.065	.022	21.993	.086	-.134	.005	
	7 Hz HFO	.070*	.019	16.383	.023	.007	.133	
Based on estimated marginal means	
*. The mean difference is significant at the .05 level.	
a. Dependent Variable: Arterial pH.	
c. Adjustment for multiple comparisons: Bonferroni.	


Pairwise Comparisons for Shunt Fractiona	
(I) Ventilatory Technique	(J) Ventilatory Technique	Mean Difference (I-J)	Std. Error	df	Sig.c	95% Confidence Interval for Differencec	
						Lower Bound	Upper Bound	
CV1	4Hz HFO	.126*	.018	24.417	.000	.070	.183	
	4Hz HFO-TGI	.111*	.019	22.077	.000	.051	.171	
	7 Hz HFO	.105*	.023	24.097	.001	.036	.175	
	CV2	-.010	.026	18.390	1.000	-.093	.074	
4Hz HFO	CV1	-.126*	.018	24.417	.000	-.183	-.070	
	4Hz HFO-TGI	-.015	.017	13.243	1.000	-.072	.041	
	7 Hz HFO	-.021	.021	16.704	1.000	-.088	.046	
	CV2	-.136*	.025	21.684	.000	-.213	-.058	
4Hz HFO-TGI	CV1	-.111*	.019	22.077	.000	-.171	-.051	
	4Hz HFO	.015	.017	13.243	1.000	-.041	.072	
	7 Hz HFO	-.006	.022	20.785	1.000	-.074	.063	
	CV2	-.120*	.026	20.539	.001	-.201	-.040	
7 Hz HFO	CV1	-.105*	.023	24.097	.001	-.175	-.036	
	4Hz HFO	.021	.021	16.704	1.000	-.046	.088	
	4Hz HFO-TGI	.006	.022	20.785	1.000	-.063	.074	
	CV2	-.115*	.028	25.434	.004	-.201	-.028	
CV2	CV1	.010	.026	18.390	1.000	-.074	.093	
	4Hz HFO	.136*	.025	21.684	.000	.058	.213	
	4Hz HFO-TGI	.120*	.026	20.539	.001	.040	.201	
	7 Hz HFO	.115*	.028	25.434	.004	.028	.201	
Based on estimated marginal means	
*. The mean difference is significant at the .05 level.	
a. Dependent Variable: Shunt Fraction.	
c. Adjustment for multiple comparisons: Bonferroni.	


Paired Samples Test	
	Paired Differences	t	df	Sig. (2-tailed)	
	Mean	Std. Deviation	Std. Error Mean	95% Confidence Interval of the Difference				
				Lower	Upper				
Pair 1	End-inspiratory Plateau Pressure1 (cmH2O) - End-inspiratory Plateau Pressure2 (cmH2O)	1.97059	1.61507	.39171	1.14020	2.80098	5.031	16	.000	
Pair 2	End-expiratory Plateau Pressure1 (cmH2O) - End-expiratory Plateau Pressure2 (cmH2O)	.44118	.99816	.24209	-.07203	.95438	1.822	16	.087	
Pair 3	Driving Pressure1 (cmH2O) - Driving Pressure2 (cmH2O)	1.52941	1.30469	.31643	.85860	2.20022	4.833	16	.000	
Pair 4	Respiratory System Compliance1 (mL/cmH2O) - Respiratory System Compliance2 (mL/cmH2O)	-6.52353	10.26709	2.49014	-11.80238	-1.24468	-2.620	16	.019	


Pairwise Comparisons for Oxygenation Indexa	
(I) Ventilatory Technique	(J) Ventilatory Technique	Mean Difference (I-J)	Std. Error	df	Sig.c	95% Confidence Interval for Differencec	
						Lower Bound	Upper Bound	
CV1	4Hz HFO	3.050*	.944	18.263	.046	.039	6.061	
	4Hz HFO-TGI	3.480*	1.001	23.728	.020	.383	6.577	
	7 Hz HFO	1.728	1.416	23.754	1.000	-2.652	6.107	
	CV2	2.087	1.094	19.286	.715	-1.379	5.552	
4Hz HFO	CV1	-3.050*	.944	18.263	.046	-6.061	-.039	
	4Hz HFO-TGI	.430	.765	12.652	1.000	-2.164	3.023	
	7 Hz HFO	-1.323	1.269	18.276	1.000	-5.370	2.725	
	CV2	-.964	.896	22.467	1.000	-3.752	1.824	
4Hz HFO-TGI	CV1	-3.480*	1.001	23.728	.020	-6.577	-.383	
	4Hz HFO	-.430	.765	12.652	1.000	-3.023	2.164	
	7 Hz HFO	-1.752	1.312	20.981	1.000	-5.866	2.361	
	CV2	-1.393	.956	21.361	1.000	-4.385	1.599	
7 Hz HFO	CV1	-1.728	1.416	23.754	1.000	-6.107	2.652	
	4Hz HFO	1.323	1.269	18.276	1.000	-2.725	5.370	
	4Hz HFO-TGI	1.752	1.312	20.981	1.000	-2.361	5.866	
	CV2	.359	1.384	22.068	1.000	-3.957	4.675	
CV2	CV1	-2.087	1.094	19.286	.715	-5.552	1.379	
	4Hz HFO	.964	.896	22.467	1.000	-1.824	3.752	
	4Hz HFO-TGI	1.393	.956	21.361	1.000	-1.599	4.385	
	7 Hz HFO	-.359	1.384	22.068	1.000	-4.675	3.957	
Based on estimated marginal means	
*. The mean difference is significant at the .05 level.	
a. Dependent Variable: Oxygenation Index.	
c. Adjustment for multiple comparisons: Bonferroni.	


Pairwise Comparisons for Mean Arterial Pressurea	
(I) Ventilatory Technique	(J) Ventilatory Technique	Mean Difference (I-J)	Std. Error	df	Sig.c	95% Confidence Interval for Differencec	
						Lower Bound	Upper Bound	
CV1	4Hz HFO	-10.138*	2.328	21.697	.003	-17.411	-2.866	
	4Hz HFO-TGI	-7.826*	1.955	20.827	.007	-13.963	-1.689	
	7 Hz HFO	-4.794	2.234	23.138	.426	-11.726	2.137	
	CV2	-3.694	1.643	19.442	.363	-8.893	1.505	
4Hz HFO	CV1	10.138*	2.328	21.697	.003	2.866	17.411	
	4Hz HFO-TGI	2.313	2.328	15.447	1.000	-5.299	9.924	
	7 Hz HFO	5.344	2.575	23.388	.491	-2.635	13.324	
	CV2	6.444	2.083	17.130	.065	-.260	13.149	
4Hz HFO-TGI	CV1	7.826*	1.955	20.827	.007	1.689	13.963	
	4Hz HFO	-2.313	2.328	15.447	1.000	-9.924	5.299	
	7 Hz HFO	3.032	2.244	21.648	1.000	-3.979	10.043	
	CV2	4.132	1.656	17.837	.226	-1.167	9.431	
7 Hz HFO	CV1	4.794	2.234	23.138	.426	-2.137	11.726	
	4Hz HFO	-5.344	2.575	23.388	.491	-13.324	2.635	
	4Hz HFO-TGI	-3.032	2.244	21.648	1.000	-10.043	3.979	
	CV2	1.100	1.978	19.765	1.000	-5.145	7.345	
CV2	CV1	3.694	1.643	19.442	.363	-1.505	8.893	
	4Hz HFO	-6.444	2.083	17.130	.065	-13.149	.260	
	4Hz HFO-TGI	-4.132	1.656	17.837	.226	-9.431	1.167	
	7 Hz HFO	-1.100	1.978	19.765	1.000	-7.345	5.145	
Based on estimated marginal means	
*. The mean difference is significant at the .05 level.	
a. Dependent Variable: Mean Arterial Pressure (mmHg).	
c. Adjustment for multiple comparisons: Bonferroni.	


Pairwise Comparisons for Heart Ratea	
(I) Ventilatory Technique	(J) Ventilatory Technique	Mean Difference (I-J)	Std. Error	df	Sig.c	95% Confidence Interval for Differencec	
						Lower Bound	Upper Bound	
CV1	4Hz HFO	-.327	3.406	21.473	1.000	-10.977	10.323	
	4Hz HFO-TGI	-.015	3.057	17.569	1.000	-9.820	9.791	
	7 Hz HFO	-10.059	3.391	23.660	.068	-20.555	.437	
	CV2	-3.176	3.246	22.187	1.000	-13.292	6.939	
4Hz HFO	CV1	.327	3.406	21.473	1.000	-10.323	10.977	
	4Hz HFO-TGI	.312	2.128	16.992	1.000	-6.545	7.170	
	7 Hz HFO	-9.732*	2.612	21.625	.012	-17.894	-1.569	
	CV2	-2.849	2.421	22.239	1.000	-10.391	4.693	
4Hz HFO-TGI	CV1	.015	3.057	17.569	1.000	-9.791	9.820	
	4Hz HFO	-.312	2.128	16.992	1.000	-7.170	6.545	
	7 Hz HFO	-10.044*	2.138	18.703	.002	-16.844	-3.244	
	CV2	-3.162	1.900	16.039	1.000	-9.338	3.015	
7 Hz HFO	CV1	10.059	3.391	23.660	.068	-.437	20.555	
	4Hz HFO	9.732*	2.612	21.625	.012	1.569	17.894	
	4Hz HFO-TGI	10.044*	2.138	18.703	.002	3.244	16.844	
	CV2	6.882	2.401	17.628	.104	-.815	14.580	
CV2	CV1	3.176	3.246	22.187	1.000	-6.939	13.292	
	4Hz HFO	2.849	2.421	22.239	1.000	-4.693	10.391	
	4Hz HFO-TGI	3.162	1.900	16.039	1.000	-3.015	9.338	
	7 Hz HFO	-6.882	2.401	17.628	.104	-14.580	.815	
Based on estimated marginal means	
*. The mean difference is significant at the .05 level.	
a. Dependent Variable: Heart Rate.	
c. Adjustment for multiple comparisons: Bonferroni.	


Pairwise Comparisons for Cardiac Indexa	
(I) Ventilatory Technique	(J) Ventilatory Technique	Mean Difference (I-J)	Std. Error	df	Sig.c	95% Confidence Interval for Differencec	
						Lower Bound	Upper Bound	
CV1	4Hz HFO	-.105	.079	16.027	1.000	-.362	.152	
	4Hz HFO-TGI	-.180	.083	18.372	.448	-.446	.086	
	7 Hz HFO	.147	.111	28.619	1.000	-.191	.485	
	CV2	-.076	.097	22.162	1.000	-.378	.225	
4Hz HFO	CV1	.105	.079	16.027	1.000	-.152	.362	
	4Hz HFO-TGI	-.075	.031	11.266	.322	-.182	.032	
	7 Hz HFO	.252	.080	15.973	.065	-.010	.514	
	CV2	.028	.059	14.393	1.000	-.167	.224	
4Hz HFO-TGI	CV1	.180	.083	18.372	.448	-.086	.446	
	4Hz HFO	.075	.031	11.266	.322	-.032	.182	
	7 Hz HFO	.327*	.085	19.068	.011	.058	.596	
	CV2	.103	.065	16.432	1.000	-.107	.314	
7 Hz HFO	CV1	-.147	.111	28.619	1.000	-.485	.191	
	4Hz HFO	-.252	.080	15.973	.065	-.514	.010	
	4Hz HFO-TGI	-.327*	.085	19.068	.011	-.596	-.058	
	CV2	-.224	.098	24.851	.313	-.525	.078	
CV2	CV1	.076	.097	22.162	1.000	-.225	.378	
	4Hz HFO	-.028	.059	14.393	1.000	-.224	.167	
	4Hz HFO-TGI	-.103	.065	16.432	1.000	-.314	.107	
	7 Hz HFO	.224	.098	24.851	.313	-.078	.525	
Based on estimated marginal means	
*. The mean difference is significant at the .05 level.	
a. Dependent Variable: Cardiac Index.	
c. Adjustment for multiple comparisons: Bonferroni.	


Pairwise Comparisons for SVIa	
(I) Ventilatory Technique	(J) Ventilatory Technique	Mean Difference (I-J)	Std. Error	df	Sig.c	95% Confidence Interval for Differencec	
						Lower Bound	Upper Bound	
CMV	HFO4HZ	-1.244	1.302	19.057	1.000	-5.376	2.887	
	HFOTGI4HZ	-2.438	1.385	22.772	.919	-6.743	1.867	
	HFO7HZ	4.765*	1.466	26.290	.032	.273	9.257	
	CMV	.006	1.304	18.237	1.000	-4.156	4.168	
HFO4HZ	CMV	1.244	1.302	19.057	1.000	-2.887	5.376	
	HFOTGI4HZ	-1.194	1.222	14.970	1.000	-5.212	2.825	
	HFO7HZ	6.009*	1.327	19.369	.002	1.809	10.209	
	CMV	1.250	1.145	24.098	1.000	-2.288	4.788	
HFOTGI4HZ	CMV	2.438	1.385	22.772	.919	-1.867	6.743	
	HFO4HZ	1.194	1.222	14.970	1.000	-2.825	5.212	
	HFO7HZ	7.203*	1.408	22.485	.000	2.821	11.584	
	CMV	2.444	1.239	19.367	.630	-1.478	6.366	
HFO7HZ	CMV	-4.765*	1.466	26.290	.032	-9.257	-.273	
	HFO4HZ	-6.009*	1.327	19.369	.002	-10.209	-1.809	
	HFOTGI4HZ	-7.203*	1.408	22.485	.000	-11.584	-2.821	
	CMV	-4.759*	1.329	18.328	.021	-8.995	-.522	
CMV	CMV	-.006	1.304	18.237	1.000	-4.168	4.156	
	HFO4HZ	-1.250	1.145	24.098	1.000	-4.788	2.288	
	HFOTGI4HZ	-2.444	1.239	19.367	.630	-6.366	1.478	
	HFO7HZ	4.759*	1.329	18.328	.021	.522	8.995	
Based on estimated marginal means	
*. The mean difference is significant at the .05 level.	
a. Dependent Variable: Stroke Volume Index.	
c. Adjustment for multiple comparisons: Bonferroni.	


Pairwise Comparisons for DO2Ia	
(I) Ventilatory Technique	(J) Ventilatory Technique	Mean Difference (I-J)	Std. Error	df	Sig.c	95% Confidence Interval for Differencec	
						Lower Bound	Upper Bound	
CV1	4Hz HFO	-37.585*	8.985	16.751	.006	-66.601	-8.569	
	4Hz HFO-TGI	-43.422*	9.466	19.259	.002	-73.412	-13.432	
	7 Hz HFO	-4.882	13.342	28.019	1.000	-45.530	35.767	
	CV2	-9.489	9.901	18.735	1.000	-40.970	21.993	
4Hz HFO	CV1	37.585*	8.985	16.751	.006	8.569	66.601	
	4Hz HFO-TGI	-5.836	4.364	12.112	1.000	-20.768	9.095	
	7 Hz HFO	32.704	10.448	15.827	.065	-1.330	66.738	
	CV2	28.097*	5.403	16.917	.001	10.673	45.520	
4Hz HFO-TGI	CV1	43.422*	9.466	19.259	.002	13.432	73.412	
	4Hz HFO	5.836	4.364	12.112	1.000	-9.095	20.768	
	7 Hz HFO	38.540*	10.864	19.005	.022	4.061	73.019	
	CV2	33.933*	6.170	17.801	.000	14.179	53.686	
7 Hz HFO	CV1	4.882	13.342	28.019	1.000	-35.767	45.530	
	4Hz HFO	-32.704	10.448	15.827	.065	-66.738	1.330	
	4Hz HFO-TGI	-38.540*	10.864	19.005	.022	-73.019	-4.061	
	CV2	-4.607	11.246	20.747	1.000	-39.914	30.700	
CV2	CV1	9.489	9.901	18.735	1.000	-21.993	40.970	
	4Hz HFO	-28.097*	5.403	16.917	.001	-45.520	-10.673	
	4Hz HFO-TGI	-33.933*	6.170	17.801	.000	-53.686	-14.179	
	7 Hz HFO	4.607	11.246	20.747	1.000	-30.700	39.914	
Based on estimated marginal means	
*. The mean difference is significant at the .05 level.	
a. Dependent Variable: Oxygen Delivery Index.	
c. Adjustment for multiple comparisons: Bonferroni.	


Pairwise Comparisonsfor VO2Ia	
(I) Ventilatory Technique	(J) Ventilatory Technique	Mean Difference (I-J)	Std. Error	df	Sig.b	95% Confidence Interval for Differenceb	
						Lower Bound	Upper Bound	
CV1	4Hz HFO	-5.104	5.578	15.808	1.000	-23.276	13.068	
	4Hz HFO-TGI	-5.185	6.036	19.779	1.000	-24.245	13.875	
	7 Hz HFO	4.859	7.299	27.393	1.000	-17.422	27.140	
	CV2	4.535	7.384	26.546	1.000	-18.069	27.139	
4Hz HFO	CV1	5.104	5.578	15.808	1.000	-13.068	23.276	
	4Hz HFO-TGI	-.081	2.333	11.923	1.000	-8.090	7.928	
	7 Hz HFO	9.963	4.812	16.121	.548	-5.667	25.592	
	CV2	9.639	4.940	15.506	.693	-6.506	25.784	
4Hz HFO-TGI	CV1	5.185	6.036	19.779	1.000	-13.875	24.245	
	4Hz HFO	.081	2.333	11.923	1.000	-7.928	8.090	
	7 Hz HFO	10.044	5.336	22.018	.731	-6.598	26.686	
	CV2	9.720	5.452	20.103	.897	-7.462	26.903	
7 Hz HFO	CV1	-4.859	7.299	27.393	1.000	-27.140	17.422	
	4Hz HFO	-9.963	4.812	16.121	.548	-25.592	5.667	
	4Hz HFO-TGI	-10.044	5.336	22.018	.731	-26.686	6.598	
	CV2	-.324	6.824	27.715	1.000	-21.134	20.487	
CV2	CV1	-4.535	7.384	26.546	1.000	-27.139	18.069	
	4Hz HFO	-9.639	4.940	15.506	.693	-25.784	6.506	
	4Hz HFO-TGI	-9.720	5.452	20.103	.897	-26.903	7.462	
	7 Hz HFO	.324	6.824	27.715	1.000	-20.487	21.134	
Based on estimated marginal means	
a. Dependent Variable: Oxygen Consumption Index.	
b. Adjustment for multiple comparisons: Bonferroni.


	

Pairwise Comparisons for SVRIa	
(I) Ventilatory Technique	(J) Ventilatory Technique	Mean Difference (I-J)	Std. Error	df	Sig.b	95% Confidence Interval for Differenceb	
						Lower Bound	Upper Bound	
CV1	4Hz HFO	-148.688	58.378	23.737	.178	-329.307	31.931	
	4Hz HFO-TGI	-57.595	53.523	18.056	1.000	-228.613	113.422	
	7 Hz HFO	-161.675	76.978	21.441	.477	-402.444	79.093	
	CV2	-39.672	57.326	13.414	1.000	-231.836	152.491	
4Hz HFO	CV1	148.688	58.378	23.737	.178	-31.931	329.307	
	4Hz HFO-TGI	91.092	37.315	5.098	.576	-84.923	267.107	
	7 Hz HFO	-12.988	67.202	21.497	1.000	-223.119	197.143	
	CV2	109.015	43.322	24.178	.189	-24.773	242.804	
4Hz HFO-TGI	CV1	57.595	53.523	18.056	1.000	-113.422	228.613	
	4Hz HFO	-91.092	37.315	5.098	.576	-267.107	84.923	
	7 Hz HFO	-104.080	63.030	17.777	1.000	-305.906	97.746	
	CV2	17.923	36.517	19.957	1.000	-97.258	133.105	
7 Hz HFO	CV1	161.675	76.978	21.441	.477	-79.093	402.444	
	4Hz HFO	12.988	67.202	21.497	1.000	-197.143	223.119	
	4Hz HFO-TGI	104.080	63.030	17.777	1.000	-97.746	305.906	
	CV2	122.003	66.290	14.228	.867	-97.821	341.827	
CV2	CV1	39.672	57.326	13.414	1.000	-152.491	231.836	
	4Hz HFO	-109.015	43.322	24.178	.189	-242.804	24.773	
	4Hz HFO-TGI	-17.923	36.517	19.957	1.000	-133.105	97.258	
	7 Hz HFO	-122.003	66.290	14.228	.867	-341.827	97.821	
Based on estimated marginal means	
a. Dependent Variable: SVRI.	
b. Adjustment for multiple comparisons: Bonferroni.	
